# Neural control and innate self-tuning of the hair cell’s active process

**DOI:** 10.1016/j.bpj.2024.09.006

**Published:** 2024-09-06

**Authors:** Charles Metzler-Winslow, Martín A. Toderi, Dolores Bozovic

**Affiliations:** 1 Department of Physics and Astronomy, University of California, Los Angeles, Los Angeles, California; 2 California NanoSystems Institute, University of California, Los Angeles, Los Angeles, California

## Abstract

We propose a model for the feedback control processes that underlie the robustness and high sensitivity of mechanosensory hair cells. Our model encompasses self-tuning active processes intrinsic to these cells, which drive the amplification of mechanical stimuli by consuming metabolic energy, and a neural input process that protects these cells from damage caused by powerful stimuli. We explore the effects of these two feedback mechanisms on mechanical self-oscillations of the sense cells and their response to external forcing.

## Significance

The sensory cells responsible for hearing rely on multiple feedback processes to fine-tune their response to sound. Intrinsic force feedback on hair bundles, the sensory organelles of these cells, underlies the extraordinary sensitivity of hearing and the spontaneous self-oscillations of hair bundles. Neural feedback further adjusts the sensitivity of hearing and the properties of hair bundle self-oscillations. We propose that intrinsic feedback can protect hair cells from damage by reducing their gain in response to loud sounds, and we show that both intrinsic feedback and neural feedback can be understood as processes that govern a parameter representing the propensity of the hair bundle to self-oscillate.

## Introduction

Auditory systems of vertebrate organisms contain biological sensors that are capable of detecting extremely weak signals. At the quiet extreme of their dynamic range, hair cells of the inner ear reliably respond to sounds that deliver less power to their sensory organelles than background noise (1). The sensory organelle of the hair cell is the hair bundle; it comprises rows of stereocilia—long, narrow cylindrical extensions of the cell membrane—arranged in a staircase-like structure that protrudes from the surface of the cell (2,3). This array of stereocilia transduces mechanical deflections into ionic currents, which trigger the transmission of information about the auditory signal to the brainstem via the auditory nerve (4,5). At the loud extreme, hair cells detect sounds 12 orders of magnitude larger in intensity than the softest audible sounds, so the auditory system must balance sensitivity to weak stimuli with protection of the delicate machinery of hair cells from damage caused by powerful signals (6,7,8,9).

### Signatures of active mechanics

To achieve its extraordinary sensitivity, the auditory system must overcome significant viscous dissipation (1,10,11). Previous studies have revealed a rich network of cellular processes that is capable of counteracting damping by consuming metabolic energy (12,13,14). These energy-consuming processes explain the paradigmatic nonlinear features of hearing: amplification of soft sounds, sharp frequency discrimination, compression of the dynamic range of sound inputs, and self-oscillations (15,16,17,18,19).

The hearing system must also anticipate future stimuli by performing sensory adaptation to maximize sensitivity and prevent damage. While the centrality of active amplification is well established, the feedback processes that control the gain of the hearing system, thus protecting hair cells from damage caused by loud sounds, remain obscure. In this work, we present a model for hair cell dynamics that captures the effects of two such processes.

### Efferent feedback

One process that has been shown to serve a protective role in hearing is efferent input. The axons of efferent neurons synapse onto the hair cell membrane, where postsynaptic receptor activation triggers hyperpolarization. This flow of information from the brain to sensory cells modulates the sensitivity of hearing. Activation of efferent neurons reduces the hearing system’s gain and blunts its frequency discrimination (for a review of efferent physiology, see (20)). When efferent neurons are severed, even sustained moderate-level stimuli permanently damage the hearing system (21).

Efferent neurons have also been shown to affect active cellular processes at the level of individual hair bundles. Activation of efferent neurons increases the threshold stimulus amplitude necessary to elicit a significant frequency-locked hair bundle response, rendering hair bundles less sensitive to applied stimuli. Furthermore, the innate active self-oscillations exhibited by hair bundles ex vivo (22) are inhibited by efferent activation.

### Intrinsic hair cell feedback

Another source of robustness in the auditory system is the network of processes that suppress spontaneous hair bundle oscillations in response to large-amplitude stimulus forcing. Following the application of strong boxcar function (23) or sinusoidal (24) forcing ex vivo, hair cells exhibited an interval of quiescence after cessation of the stimulus before recovering oscillatory behavior, and the length of the quiescent interval increased with the duration of forcing. This effect was observed in biological preparations in which efferent neurons had been severed, so it indicates an intrinsic feedback process in hair cells that does not rely on neural feedback.

## Materials and methods

We develop a simple theoretical model for the multifaceted feedback processes that regulate the response of auditory hair cells. Our nonlinear, continuous-time, deterministic model includes an innate, active self-tuning process described by a feedback differential equation governing a parameter that determines the propensity of the hair bundle to self-oscillate. We represent efferent input as parametric forcing that controls this parameter. Although our model is simple and requires few assumptions, it captures many intricate properties of the feedback phenomena that shape the response of auditory hair cells.

Innate spontaneous oscillations, amplification, dynamic range compression, and sharp frequency response are distinctive features of a dynamical system operating in the vicinity of a Hopf bifurcation. These bifurcations, which mark qualitative changes from quiescence to self-oscillation, have been identified in detailed biophysical models for hair bundle motion (2,14,25). The paradigmatic attributes of hair bundle motion are captured parsimoniously by a differential equation that is universal around any equilibrium that undergoes a Hopf bifurcation (26,27). In the most simple choice of coordinates, which can be attained via transformations dictated by normal form theory, this equation is called the Stuart-Landau equation:

(1) $$\frac{dz}{dt}=\left(\mu_{c}+\mu \right)z+i\omega z+\beta {\left|z\right|}^{2}z\text{.}$$

The complex variable z(t)=x(t)+iy(t) represents a state function with a fixed point z=0. The real part x(t) represents the instantaneous position of the hair bundle along the line spanned by its height gradient. The control parameter *μ* represents the displacement from the supercritical Hopf bifurcation point. A transition in the stability of the fixed point z=0 and the geometry of the orbits of Eq. 1 occurs across the bifurcation point $\mu_{c}$. For values of the control parameter below the critical threshold value, $\mu <-\mu_{c}$, solutions to Eq. 1 decay to a stable node at z=0. For values above the critical value, $\mu >-\mu_{c}$, the fixed point z=0 loses stability, and solutions approach a stable limit cycle. The parameter *ω* represents the angular frequency of limit cycle oscillation approached as $\mu \to -\mu_{c}^{+}$, and the parameter $\beta =b^{\prime }+ib^{\prime \prime }$ determines the strength of the nonlinearity. The real part $b^{\prime }$ governs the rate of growth of limit cycle oscillation amplitude A=|z| as a function of the control parameter according to the amplitude equation:

(2) $$A=\sqrt{-\frac{\left(\mu_{c}+\mu \right)}{b^{\prime }}.}$$

The imaginary part $b^{\prime \prime }$ dictates the degree of coupling between the amplitude *A* and the angular frequency $\dot{\phi }=d_{t}\;\arg\left(z\right)$ according to the frequency equation:

(3) $$\dot{\phi }=\omega +b^{\prime \prime }A^{2}\text{.}$$

When the amplitude and frequency are independent, $b^{\prime \prime }=0$, the oscillator is termed isochronous.

### Response to external forcing

We represent stimulus using an additive forcing function $F_{a}\left(t\right)$. Including this external stimulus, the governing equation becomes

(4) $$\frac{dz}{dt}=\left(\mu_{c}+\mu +i\omega +\beta {\left|z\right|}^{2}\right)z+F_{a}\text{.}$$

For a pure tone stimulus applied at the characteristic frequency of the oscillator $F_{a}\left(t\right)=Fe^{i\omega t}$, the response amplitude at the bifurcation point is given by a one-third power law:

(5) $$A=-b^{\prime -1/3}F^{1/3}\text{.}$$

Hence, the gain characterizing sensitivity diverges in the F→0 limit, $dA/dF=b^{\prime -1/3}F^{-2/3}/3$. For forcing frequencies sufficiently far from *ω*, *A* is linear in *F*. A one-third power law for the resonant response has been observed ex vivo for external forcing amplitudes spanning much of the total physiologically relevant range (28).

Considering stimulus frequencies on each side of the characteristic frequency, the width at half-maximum of the amplitude function $A\left(\omega^{\prime },F\right)$ decreases with decreasing stimulus amplitude:

(6) $$\Delta \omega_{A_{\max}/2}^{\prime }\propto F^{2/3}\text{.}$$

The width of response has been experimentally shown to increase with the strength of forcing. Hence, small-amplitude stimuli induce narrowly tuned high-gain responses around the characteristic frequency, while large-amplitude stimuli induce broadly tuned low-gain responses (26). These response properties differ sharply from those of passive resonating systems such as the driven damped harmonic oscillator, for which the response width and gain are both independent of forcing amplitude at all forcing frequencies.

### Critical self-tuning

We include a self-tuning equation that automatically maintains the system in the proximity of the critical point $\mu =-\mu_{c}$. Since the one-third power law for the response amplitude holds at the critical point, while the response is linear away from the critical point for sufficiently small forcing amplitudes, such a feedback control process would maintain the high sensitivity of hair cells to soft sounds and ensure a wide dynamic range (26). A system that self-tunes to the vicinity of a Hopf bifurcation, like the one we study here, is termed a critical oscillator (8,29,30,31,32).

In anamniote species and some amniotes, the intrinsic cellular processes underlying sensitivity and spontaneous oscillation have been localized to the hair bundle. These active mechanisms involve the movement of myosin motors and changes to the probability that transduction channels will close (25). Earlier work has indicated that ${\text{Ca}}^{2+}$ ions, which flow into stereocilia as part of the hair cell transduction current, serve as a crucial regulator of active hair cell mechanisms. The effects of ${\text{Ca}}^{2+}$ on these active mechanisms can be broadly classified as negative feedback: ${\text{Ca}}^{2+}$ reduces the force exerted by myosin motors and makes transduction channels more likely to close (25,33).

We note that a different set of active mechanisms are thought to underlie sensitivity in the hair cells of mammalian organisms (34,35). We emphasize that the abstract representation of sensitivity in the universal framework based on the Stuart-Landau equation is independent of the particular active mechanisms underlying sensitivity.

We interpret the control parameter as a measure of the degree of intrinsic hair cell activity (see Eq. 2). Assuming the degree of activity is negatively affected by ${\text{Ca}}^{2+}$, we employ a specific self-tuning equation introduced in (8):

(7) $$\frac{d\mu }{dt}=-\frac{\mu }{\tau }-\frac{\alpha }{1+e^{-\gamma x}}\text{.}$$

The first term on the right-hand side of Eq. 7 produces relaxation to the value of the second term with a time constant *τ*. The second term represents the effect of ${\text{Ca}}^{2+}$ influx on the control parameter. This term follows from a simplified model of transduction channels and tip links, which are elastic protein strands that connect the transduction channel complex in each stereocilium to the side of the tallest neighboring stereocilium; each tip link is modeled as a Hookean spring connected to a transduction channel that can only occupy either a closed or open state. A canonical ensemble of such pairs of tip links and channels yields an open state probability $P_{o}$ that is a logistic function of the bundle displacement *x*. Thus, the middle term $\alpha P_{o}\left(\gamma ,x\right)$ represents the effect of the total influx of ${\text{Ca}}^{2+}$ on the degree of hair cell activity, where *α* represents the strength of the effect and is proportional to the constant influx through a single open channel multiplied by the total number of channels. At constant temperature, the parameter *γ* is proportional to the force associated with the transition from the closed to open channel state.

(36) argues that, for a large collection of systems, a control feedback law of the form $\dot{\mu }=f\left(A\right)-g\left(\mu \right)$ maintains *μ* near the critical point $-\mu_{c}$ if *f* and *g* satisfy the conditions that 1) *f* is a decreasing function of amplitude *A*, 2) *g* is an increasing function, and 3) there exists a value $A_{0}$ for which $\dot{\mu }=0$ at the critical point, $f\left(A_{0}\right)=g\left(-\mu_{c}\right)$. Eq. 7 satisfies those conditions and, indeed, dictates that the steady-state time average ⟨μ⟩ grows to approximately −ατ/2, a value near the critical point $-\mu_{c}$, in the absence of forcing when $\mu_{c}<\tau \alpha$.

### Parametric forcing

We introduce a parametric forcing function $F_{p}\left(t\right)$ to account for the effects of efferent input on hair bundle mechanics. We note that parametric forcing has been applied previously to the study of the auditory system; it provides a simple theoretical framework for describing a complex network of feedback processes (37).

While the biophysical mechanisms that produce the effects of efferent input on hair bundle mechanics remain unknown, prior work has suggested that the effects are mediated by calcium ions. The influx of ${\text{Ca}}^{2+}$ into stereocilia is controlled by its transmembrane concentration gradient and the transmembrane gradient of electric potential. Since efferent input hyperpolarizes hair cells, it increases the force driving ${\text{Ca}}^{2+}$ influx through the transduction channels. Hence, by modulating the influx of ${\text{Ca}}^{2+}$, efferent activation exerts an effect on hair bundle mechanics via the active mechanisms internal to hair cells.

When strong boxcar forcing was combined with efferent input, efferent feedback supplanted intrinsic feedback. Following cessation of large-amplitude constant mechanical stimulus, efferent nerve fiber activation caused a dramatic decrease in the length of the quiescent interval (the recovery of oscillations was very rapid when and only when efferent nerve fibers were activated) (38). Inspired by this experimental observation, we hypothesize that strong, persistent efferent synaptic activity, which we model using a constant parametric forcing term, overwhelms the internal cell dynamics responsible for the transition to quiescence and recovery of oscillations. We modify the governing equation so that $F_{p}$ replaces the control parameter *μ*:

(8) $$\frac{dz}{dt}=\left(\mu_{c}+\mu 1_{F_{p}=0}+F_{p}\right)z+i\omega z+\beta {\left|z\right|}^{2}z+F_{a}\text{.}$$

Here, 1 denotes the indicator function, and we emphasize that the first term in Eq. 8 reflects the hypothesis that efferent activation supplants intrinsic feedback: the parametric forcing function $F_{p}\left(t\right)$ replaces μ(t) when $\left|F_{p}\right|>0$.

### Experimental methods

Sacculi from the North American bullfrog (*Rana catesbeiana*) were dissected maintaining their full physiological integrity (39). Hair cells were imaged ex vivo during spontaneous oscillations of the hair bundle and electrical stimulation of the eighth cranial nerve (38). These perturbations served to trigger or modulate mechanical motions of the hair bundle. Optical imaging was then used to detect fluctuations in the light intensity of pixels, serving to determine the hair bundle position over time at the upper limit transverse sectioning plane.

Frogs of either gender were anesthetized (pentobarbital: 150 mg/kg), pithed, and decapitated following protocols approved by the University of California, Los Angeles Chancellor’s Animals Research Committee. Sacculi were excised from the inner ears of the animals and placed in oxygenated artificial perilymph solution (in mM as follows: 110 Na^+^, 2 K^+^, 1.5 Ca^2^^+^, 113 Cl^−^, 3 D-(+)-glucose, 1 Na^+^ pyruvate, 1 creatine, 5 HEPES). The epithelium was mounted in a two-compartment chamber, emulating the fluid partitioning of the in vivo physiological conditions. In this arrangement, apical surfaces were bathed in artificial endolymph (in mM as follows: 2 Na^+^, 118 K^+^, 0.25 Ca^2+^, 118 Cl^−^, 3 D-(+)-glucose, 5 HEPES) and basolateral membranes in perilymph (40). In order to allow direct mechanical access to the hair bundles, the otolithic membrane was carefully removed from the epithelium after an 8 min enzymatic dissociation with 15 g/mL collagenase IV (Sigma-Aldrich, St. Louis, MO, USA).

Recordings were performed using an upright optical microscope (Olympus BX51WI, Tokyo, Japan) with a water-immersion objective (Olympus LUMPlanFL *N* 60×, NA: 1.00) mounted on an optical table (Technical Manufacturing, Durham, CT, USA). The setup was constructed inside an acoustically isolated chamber (Industrial Acoustics, Naperville, IL, USA) to avoid introducing external perturbations to the highly sensitive hair cells. 16 bit TIFF images at a resolution of 108.3 nm/px were recorded with a high-speed camera (ORCA-Flash4.0 CMOS) at 1000 frames per second. Motion of the hair bundles was tracked using custom-made MATLAB scripts. Specifically, the centroid of the hair bundle position was determined from the two-dimensional light intensity profile in each frame of the recording. Plots of bundle position over time then provided traces of its motion.

Electrical signals were applied to the eighth cranial nerve, producing alterations of the spontaneous oscillatory pattern of the hair cells. Hair bundles oscillated with a noticeably higher frequency during activation of the efferent neurons than without stimulation. Efferent stimulation was performed with a bipolar suction electrode (A-M Systems, Sequim, WA, USA). The saccular nerve was pulled into a 0.5-mm-diameter silicon tube filled with perilymph, which was electrically connected to the positive terminal of the electrode, while the reference terminal was immersed in the perilymph compartment of the chamber. A linear stimulus isolator (World Precision Instruments A395, Sarasota, FL, USA) provided current to the suction electrode, and stimulus protocols were sent to the isolator via LabView (National Instruments, Austin, TX, USA). Constant step shaped stimuli were applied with current intensities of 50, 100, 150, and 200 *μ*A for 1 s, preceded and followed by 2 s of no stimulation. Altered bundle oscillations were observed immediately after the onset of the stimulus and ceased upon its termination. Tracking the hair bundle movement allowed us to confirm proper activation of the efferent pathway and the induced changes in hair bundle motility observed in prior literature (22).

## Results

**Figure 1:**
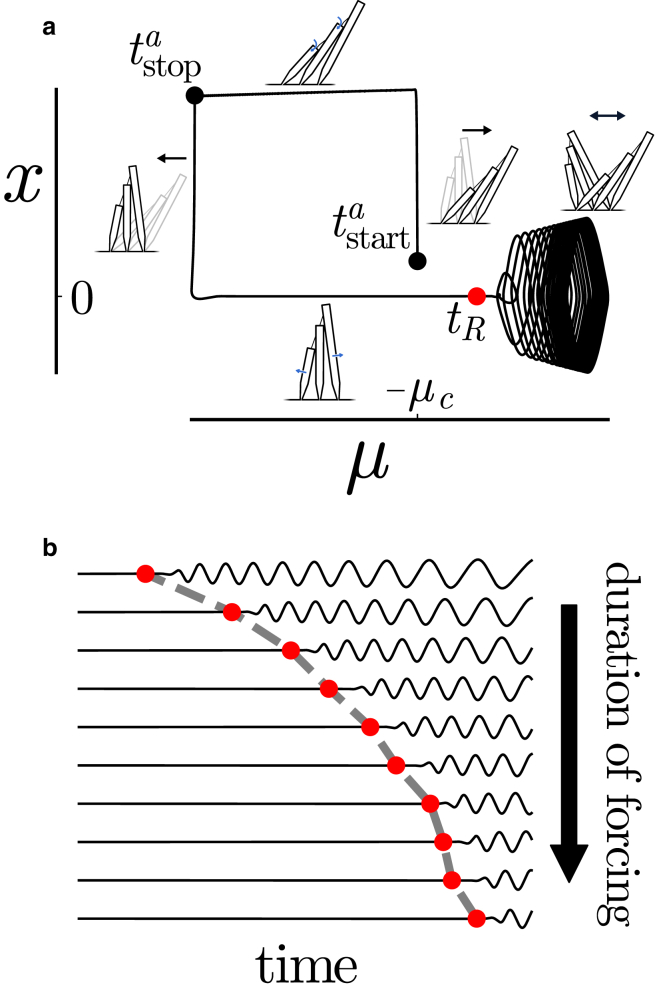
Self-tuning mediates the transition to quiescence and recovery of oscillations in response to strong constant external stimulus. (*a*) A large additive boxcar force $F_{a}=\left[\Theta \left(t-t_{\text{start}}^{a}\right)-\Theta \left(t-t_{\text{stop}}^{a}\right)\right]F$ displaces the hair bundle to a maximal position. The hair bundle position then gradually relaxes. The deflection triggers feedback on the control parameter *μ*, which decreases during external forcing and reaches a value on the quiescent side of the critical point, $\mu \left(t_{\text{stop}}^{a}\right)<-\mu_{c}$. After forcing stops, the bundle returns to its resting position and remains quiescent, while the self-tuning mechanism restores *μ* to its steady-state value. Oscillations recover at time $t_{R}$ after *μ* crosses the critical point, and the amplitude and frequency of oscillation approach their respective steady-state values asymptotically. Black arrows represent displacements, and blue arrows represent ion fluxes (specifically, *blue arrows* denote the transduction current in the upper schematic and clearance via extrusion pumps in the lower schematic). (*b*) The duration preceding recovery of spontaneous oscillations following large constant additive forcing is an increasing function of the duration of forcing. The position $x\left(t>t_{\text{stop}}^{a}\right)$ (*black curves*) is shown for a collection of forcing durations from $t_{\text{stop}}^{a}-t_{\text{start}}^{a}=40$ (*top*) to 97.5 (*bottom*). Recovery times $t_{R}$ are shown in red. For these simulations, the amplitude F=1000 and the time constant τ=35.

We suggest that the suppression and recovery of oscillations following strong constant mechanical stimulation are explained by the self-tuning dynamics of the control parameter μ(t): during stimulation, the influx term in Eq. 7 drives *μ* below the critical point, $\mu <-\mu_{c}$, where the system is quiescent. After stimulation, the control parameter returns to the oscillatory side of the critical point, $\mu >-\mu_{c}$, and settles into steady-state oscillation as shown in Fig. 1 *a*. This description is consistent with the observation that the length of the induced quiescent interval preceding the recovery of oscillations is an increasing function of stimulus duration: during stimulation, the displacement of μ(t) from the critical point increases with time. The recovery time is shown as a function of forcing duration in Fig. 1 *b*.

**Figure 2:**
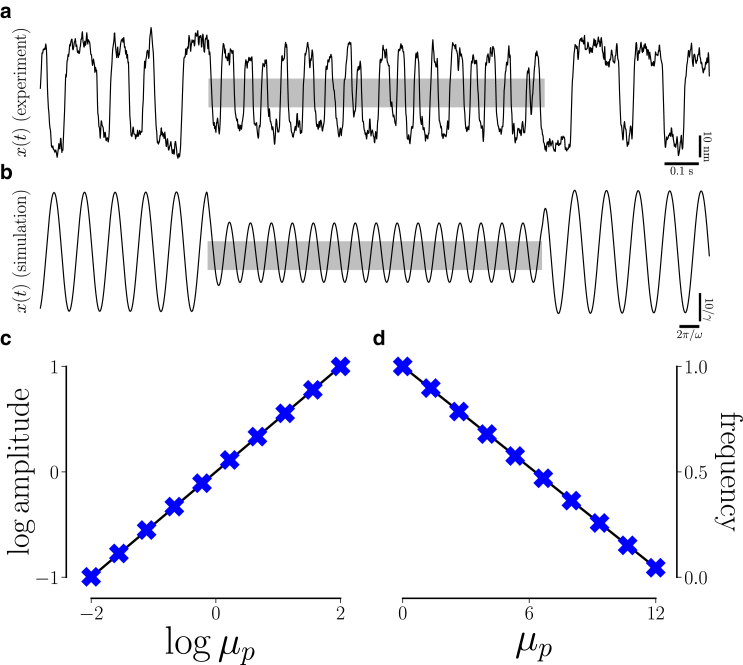
Parametric forcing reduces the amplitude and increases the frequency of spontaneous oscillations. (*a*) The hair bundle position x(t) during spontaneous oscillations obtained via microscopic imaging. Efferent neurons were electrically activated during the interval spanned by the gray rectangle. (*b*) The position x(t) obtained via numerical simulation with rectangular parametric forcing $F_{p}=\left[\Theta \left(t-t_{\text{start}}^{p}\right)-\Theta \left(t-t_{\text{stop}}^{p}\right)\right]\left(1-\mu_{c}\right)$. During the interval shown in gray, $\left[t_{\text{start}}^{p},t_{\text{stop}}^{p}\right]$, the effects of parametric forcing match the experimentally observed effects of efferent activation on spontaneous oscillations. (*c* and *d*) Predicted steady-state amplitude of oscillation $\left\langle A\right\rangle =\sqrt{-\mu_{p}/b^{\prime }}$ and frequency $\left\langle \dot{\phi }\right\rangle =\omega_{0}-\left(b^{\prime \prime }/b^{\prime }\right)\mu_{p}$ (*black solid curves*). The empirical average was taken over 50 steady-state cycles of amplitude function A(t)=|z(t)| and frequency function $\dot{\phi }\left(t\right)=d_{t}\;\arg\left(z\right)$ (*symbol x*) obtained via numerical simulation with $F_{p}=\mu_{p}-\mu_{c}$ for the entire stimulation duration.

We find that parametric forcing explains the effects of efferent activity on spontaneous oscillations. For strong, persistent efferent activity, we introduce the parametric forcing function $F_{p}=\left(\mu_{p}-\mu_{c}\right)\left[\Theta \left(t-t_{\text{start}}^{p}\right)-\Theta \left(t-t_{\text{stop}}^{p}\right)\right]$, where Θ denotes the Heaviside step function and the forcing amplitude $\mu_{p}<\mu_{c}+\left\langle \mu \right\rangle {|}_{F_{p}=0}$ (see Table 1). Consistent with experimental observations (22), this forcing reduces the amplitude and increases the frequency of spontaneous oscillation (Fig. 2). These effects are consistent with the effects of hyperpolarization of the hair cell membrane via the voltage clamp method, reinforcing the view that the central consequence of efferent activity is hyperpolarization of the cell soma (41,42).

**Table 1:** Parameter values unless otherwise specified

Parameter	Definition	Value
$\mu_{c}$	bifurcation point	20
*ω*	characteristic angular frequency	2π
*β*	strength of nonlinearity	−1−i/2
*τ*	relaxation time	10
γ	sensitivity of self-tuning	10
*α*	strength of self-tuning	3

**Figure 3:**
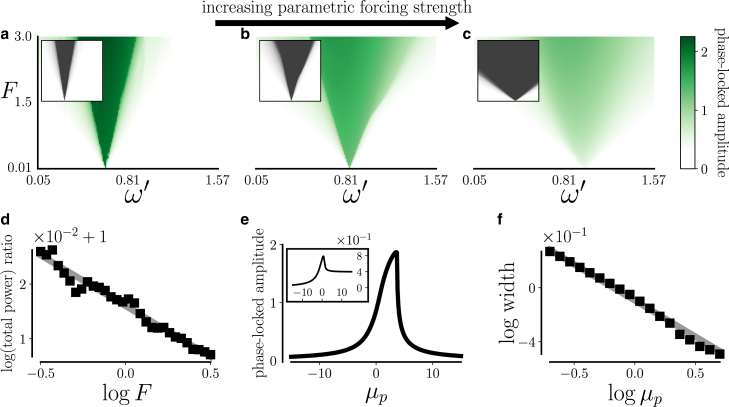
Constant parametric forcing $F_{p}=\mu_{p}-\mu_{c}$ changes the nonlinear response of a Hopf oscillator to sinusoidal additive forcing $F_{a}\left(t\right)=Fe^{i\omega^{\prime }t}\text{.}$ (*a*–*c*) The region of significant phase-locked amplitudes $A\left(\omega^{\prime }\right)=\left|F\left(z\left(t\right)\right)\right|\left(\omega^{\prime }\right)$ shifts toward larger forcing amplitudes and spreads out across forcing frequencies with increasing parametric forcing strength $S=\mu_{p}^{-1}$. (*a*) represents the case of no parametric forcing, (*b*) parametric forcing with strength S=2/5, and (*c*) parametric forcing with S=32/5. (*a*–*c*) Insets: the vector strength V, an estimate of the coherence function between x(t) and $F_{a}\left(t\right)$, is shown in gray (frequency and amplitude axes identical between *images* and *insets*). $V=\left|\left\langle e^{i\left(\phi -\phi^{\prime }\right)}\right\rangle \right|$, where ϕ(t) represents the angular displacement of *z* and $\phi^{\prime }=\omega^{\prime }t\text{.}$ (*d*) Ratio *R* of total logarithmic powers, $R=\log_{10}\Sigma_{\omega^{\prime }}P_{\text{inactive}}\left(\omega^{\prime }\right)/\log_{10}\Sigma_{\omega^{\prime }}P_{\text{moderate}}\left(\omega^{\prime }\right)$, where $P_{\text{inactive}}$ and $P_{\text{moderate}}$ denote power spectral densities of x(t) estimated using Bartlett’s method with $\mu_{p}=0$ and $\mu_{p}=5/2$, respectively. In this case, $R\approx 1+\log_{10}F^{-0.019}$. (*e*) Phase-locked amplitudes to forcing with F=1 and $\omega^{\prime }=8\pi /5$ are shown as a function of the amplitude of parametric forcing of a Hopf oscillator in the isochronous and nonisochronous case, β=−1 (*inset*) and β=−1−i/2 (*image*) respectively. We note that for both the isochronous and nonisochronous oscillators, the amplitude $A\left(\mu_{p}\right)$ increases between the bifurcation point $\mu_{p}=0$ and the point of maximal response, ${\mathrm{argmax}}_{\mu_{p}}\left[A\right]>0$, so phase-locked responses are weaker closer to the bifurcation point on the oscillatory side. (*f*) Width at half-maximum $\Delta \omega_{V_{\max}/2}^{\prime }$ of the vector strength for forcing amplitude F=1.5 as a function of parametric forcing amplitude $\mu_{p}$. Here, $\Delta \omega_{V_{\max}/2}^{\prime }\approx -9.5\times 10^{-2}+\mu_{p}^{-0.54}$.

Hair bundles have been shown to phase lock to sinusoidal forcing within a range of forcing frequencies around their frequency of spontaneous oscillation. The region of amplitudes and frequencies over which phase locking occurs is called the Arnold tongue. Consistent with the experimental observation that the response amplitudes over a hair bundle Arnold tongue were vastly reduced by efferent activity (22), constant parametric forcing decreased the phase-locked response amplitudes over the critical oscillator’s Arnold tongue. This can be seen in Fig. 3, *a*–*c*, which depicts the forcing frequency component of the oscillator’s magnitude spectrum. The insets of Fig. 3, *a*–*c*, depict the degree of phase synchronization over the Arnold tongue. The inset Arnold tongues widened in response to increasing parametric forcing strength, which we define as $S=1/\mu_{p}$, so parametric forcing blunted the oscillator’s frequency selectivity. The rate of widening is shown for a particular amplitude of external forcing in Fig. 3 *f*. We note that the reduction in the total phase-locked response as a result of parametric forcing—as illustrated in Fig. 3 *d*—is more dramatic for soft sounds than loud sounds.

The phase-locked response amplitude for off-resonance external forcing is shown as a function of parametric forcing amplitude in Fig. 3 *e*. On the quiescent side of the bifurcation point, the position function x(t) is completely entrained to the external forcing. Close to the bifurcation on the oscillatory side, the oscillator remains entrained, and the intrinsic energy quantified by $\mu_{p}$ contributes to the phase-locked response amplitude. When the parametric forcing amplitude exceeds a threshold value, harmonics of the forcing frequency emerge in the oscillator response, and the phase-locked response amplitude decreases. For sufficiently large values of $\mu_{p}$, the limit cycle frequency mode dominates.

**Figure 4:**
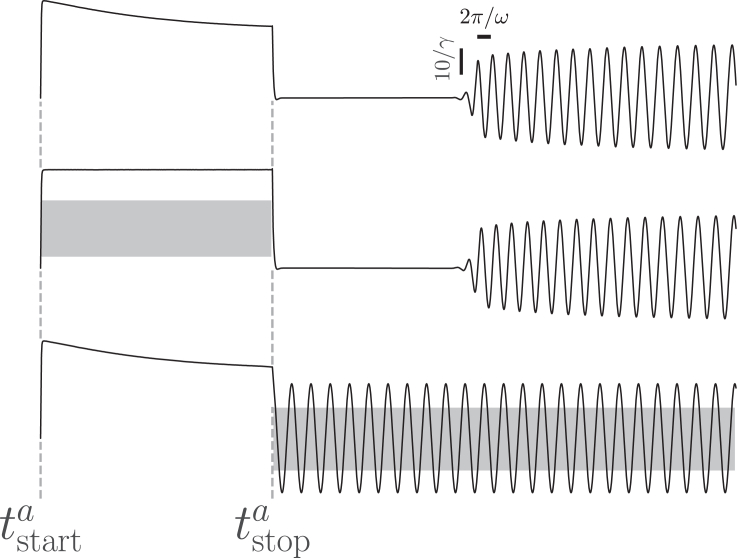
Parametric forcing causes the time before recovery of oscillations after large-amplitude forcing to vanish, and the application of parametric forcing during the interval following large-amplitude forcing is sufficient for the recovery of oscillations. Position x(t) in response to overstimulating additive forcing $F_{a}=50\left[\Theta \left(t-t_{\text{start}}^{a}\right)-\Theta \left(t-t_{\text{stop}}^{a}\right)\right]$ with no parametric forcing (*top*) parametric forcing $F_{p}\left(t\right)=\left(5-\mu_{c}\right)\left[\Theta \left(t-t_{\text{start}}^{p}\right)-\Theta \left(t-t_{\text{stop}}^{p}\right)\right]$ applied only during overstimulating forcing (*middle*, interval $\left[t_{\text{start}}^{p},t_{\text{stop}}^{p}\right]=\left[t_{\text{start}}^{a},t_{\text{stop}}^{a}\right]$ shown in *gray*) and parametric forcing $F_{p}\left(t\right)$ applied after the cessation of overstimulating forcing until the end of the simulation at $t=t_{\text{end}}$ (*bottom*, interval $\left[t_{\text{start}}^{p},t_{\text{stop}}^{p}\right]=\left[t_{\text{stop}}^{a},t_{\text{end}}\right]$ shown in *gray*).

Our model reproduces the experimental observation (38) that efferent activity can eliminate the transition to and recovery from quiescence induced by strong constant mechanical stimulation (Fig. 4). Oscillations recover immediately following large-amplitude constant external forcing when, and only when, parametric forcing is applied. In contrast, when parametric forcing is applied concurrently with external forcing, it does not affect the length of the quiescent interval since the control parameter μ(t) and the parametric forcing function $F_{p}\left(t\right)$ are independent.

## Discussion

We developed a theoretical model for the effects of internal self-tuning feedback and efferent input on active hair bundle dynamics. The model captures modulation of the amplitude and frequency of spontaneous oscillations by efferent activity and the reduced sensitivity of a hair cell to sinusoidal mechanical stimulus. We note that this reduction in sensitivity might reflect the protective role of efferent input. The model also reproduces the interaction between self-tuning and efferent activity in the context of the response to large-amplitude forcing. We emphasize that the elements of this model are generic and simple: a dynamical system defined by a universal equation for motion near a transition between quiescent and self-oscillatory states, dynamic feedback that automatically poises that system at the threshold of its transition, and parametric forcing. With these three elements, we recover the effects of signals flowing from the brain to hair cells in shaping active hair bundle mechanics.

Earlier theoretical and experimental works have examined various quantities that mediate a transition between the quiescent and oscillatory states of hair bundle motion. These quantities include the hair cell membrane potential, the stiffness of stereociliary pivots, the stiffness of an elastic load on the hair bundle, the extracellular concentration of ${\text{Ca}}^{2+}$ near the tips of stereocilia, and the intracellular ${\text{Ca}}^{2+}$ concentration. While each of these quantities constitutes a candidate for the control parameter, we note that the identity of the single control parameter *μ* remains uncertain (40,41,42,43).

Specific models of hair bundle mechanics include biophysical mechanisms for the regulation of the active network of intrinsic processes underlying spontaneous oscillations and the sensitivity of detection (25). The effects of ${\text{Ca}}^{2+}$ concentration on the amplitude and frequency of spontaneous oscillations predicted by these models are consistent with our description of feedback on the control parameter *μ*. Hence, our findings are compatible with the view that the qualitative dynamic state of a hair bundle defined by the internal control parameter *μ* is affected by ${\text{Ca}}^{2+}$.

Although we focused on the impact of intrinsic and efferent feedback on hair bundle motion, our model aimed for generality, and we speculate that our model might provide theoretical insight into inhibitory feedback processes of sense cells in other systems. We suggest that parametric forcing of a quantity controlling gain can capture the key features of the modulation of cellular feedback processes that underlie sensitivity. Also, the self-tuning Eq. 7 contains elements that are likely applicable to many biological systems that contain self-regulation mechanisms mediated by ion concentrations: 1) relaxation dynamics and 2) influx through transduction channels.

Because recent experimental evidence indicates that efferent inputs to hair cells strongly influence their sensitivity and mechanics, we argue that theoretical models that aim to capture the full range of properties of the auditory sensory system should include efferent neural feedback.

## Acknowledgments

We thank Shenshen Wang and Mason Porter for useful discussions, and we thank members of D.B.’s research group for constructive comments on the manuscript. This work was funded in part by the National Science Foundation Physics of Living Systems under grant 2210316 and in part by the 10.13039/100000181Air Force Office of Scientific Research under grant FA9550-23-1-0713.

## Author contributions

D.B. and C.M.-W. designed the research. C.M.-W. developed the theoretical framework, wrote simulation code, performed analytical calculations, and ran simulations. M.A.T. contributed experimental data and wrote the experimental methods section, and C.M.-W. and D.B. wrote the article.

## Declaration of interests

The authors declare no competing interests.

## Supporting material

Document S1. Supporting materials and methods and Figures S1–S10

Document S2. Article plus supporting material
