# Supplementary material for: Neural control and innate self-tuning of the hair cell’s active process
Source: Biophys J. 2024 Sep 6;123(20):3550–7. doi: 10.1016/j.bpj.2024.09.006 (PMC11494480; doi:10.1016/j.bpj.2024.09.006)
Supplement: Document S1. Supporting materials and methods and Figures S1–S10 [file mmc1.pdf]

Biophysical Journal, Volume *123*

Supplemental information

Neural control and innate self-tuning of the hair cell's active process

Charles Metzler-Winslow, Martín A. Toderi, and Dolores Bozovic

1 Numerical Simulation Methods

We used the Dormand–Prince method to integrate a discrete-time version of equations 7 and 8 in the main text, with step size $h = 10^{-3}$ time units. For subfigure 3(e) of the main text, we used a variable order, variable step size backward-differentiation formula method to integrate equations 7 and 8.

1.1 Recovery Time

We defined the recovery time t_R as the earliest time after suppression of oscillations at which the sign of either $x(t) - x_{\text{thresh}}$ or $x(t) + x_{\text{thresh}}$ changes for the small threshold displacement parameter $x_{\text{thresh}} = 0.05$. For the recovery from a negative initial value $\mu(0) = \mu_0$, we estimate the recovery time as:

$$t_R(\mu_0) \approx \tau \log(\frac{\mu_0 + \tau(\frac{\alpha}{2} - C)}{\tau(\frac{\alpha}{2} - C)}) \tag{1}$$

The recovery time t_R is shown as a function of the initial value μ_0 in figure 1. For the recovery from a negative value of μ induced through self-tuning by constant additive forcing with duration D, we estimate the recovery time:

$$t_R(D) \approx \tau \log[\frac{e^{-D/\tau}(\alpha - C) - \alpha/2}{\alpha/2 - C}] + t_0 \tag{2}$$

where $t_0 = 7.978$ represents a constant initial time displacement. The recovery time t_R is shown as a function of the constant additive forcing duration D in figure 2.

1.2 Phase-Locked Amplitude

We estimated the phase-locked amplitudes $\mathcal{A} = |\mathcal{F}[x(t)]|(\omega')$ using the discrete Fourier transform, $\mathcal{A}_{\text{numerical}} = |\mathcal{F}_{\text{discrete}}[x(t)]|(\omega_{\text{nearest}})$, where ω_{nearest} denotes the discrete Fourier spectrum frequency closest to the forcing frequency ω'.

1.3 Angular Displacement

We estimated the angular displacement of z as the cumulative sum of the sequence of differences $\mathrm{d}\phi_i = \arg(z(t_{i+1})) - \arg(z(t_i))$ for time step index i.

1.4 Total Power

We estimated the power spectral densities $P[x(t)](\omega')$ using Bartlett's method, $P_{\text{Bartlett}}[x(t)](\omega_{\text{nearest}})$.

1.5 Full Widths at Half Maximum

We estimated the widths of the vector strengths $\mathcal{V} = |\langle e^{i(\phi - \phi')} \rangle_{\text{empirical}}|$ using the `peak_widths` function from the SciPy library. That function accepted the frequency of the peak of the vector strength, ω_{peak}, and executed the following algorithm:

1. Calculate the minimal values $\mathcal{V}^-_{\text{min}}$ and $\mathcal{V}^+_{\text{min}}$ of the vector strength on the intervals $[\omega_-, \omega_{\text{peak}}]$ and $[\omega_+, \omega_{\text{peak}}]$ where $\omega_\pm$ represent the frequencies of the closest peak with a larger height than $\mathcal{V}(\omega_{\text{peak}})$ below and above ω_{peak}, respectively, or the minimum and maximum frequencies if such peaks do not exist.

2. Calculate the peak prominence $P = \mathcal{V}(\omega_{\text{peak}}) - \max(\mathcal{V}^-_{\text{min}}, \mathcal{V}^+_{\text{min}})$.

3. Calculate the evaluation height $\mathcal{V}_{\text{eval}} = \mathcal{V}(\omega_{\text{peak}}) - 0.5 \cdot P$.

4. Calculate the frequencies $\omega^\pm_{\text{eval}}$ at which $\mathcal{V}(\omega^\pm_{\text{eval}}) = \mathcal{V}_{\text{eval}}$ using linear interpolation between the closest points of $\mathcal{V}(\omega')$ above and below $\mathcal{V}_{\text{eval}}$ on each side of the peak.

5. Return the width $\Delta\omega'_{\mathcal{V}_{\max/2}} = \omega^+_{\text{eval}} - \omega^-_{\text{eval}}$.

2 Choice of Parameter Values

The values of the fixed parameters ω and β that appear in equation 8 in the main text, as well as the parameters τ, α, and C in equation 7, affect the bundle state and control parameter dynamics significantly. The ranges of acceptable values for these parameters are constrained by conditions imposed by the modeling objectives.

The ranges of values that the parameters α and C can take are constrained by the condition that the steady state value of $\mu(t)$ must be positive in the case of zero additive forcing (so that spontaneous oscillations occur) and negative in the case of strong additive forcing (so that spontaneous oscillations are suppressed.) In the zero forcing case, the approximate steady state value of μ is $\tau(C - \frac{\alpha}{2})$, and the approximate value during overstimulating forcing is $\tau(C-\alpha)$, so the constraint is $\alpha/2 < C < \alpha$.

The intrinsic frequency ω and nonlinear parameter $\beta = b' + ib''$ are connected to the parameters τ, C, and α through the condition that the spontaneous oscillation frequency is positive, $\mathrm{d}_t \arctan(y/x) > 0$. The approximate steady-state value of the oscillation frequency is $\omega - (b''/b')\mu_{\text{steady-state}}$, so the constraint on the intrinsic frequency is $\omega > \tau b''(C - \frac{\alpha}{2})/b'$.

3 Parametric Forcing of Spontaneous Oscillations

The parametric forcing function $F_p(t)$ and control parameter term $\mu \mathbf{1}_{F_p \leq 0}(t)$ are shown in figure 3 for the case of zero additive forcing, $F_a = 0$. Since the control parameter self-tuning equation imposes negative regulation on μ as a function of position $x(t)$, the steady-state value of the control parameter is larger immediately after the cessation of parametric forcing. An example of the negative effect of parametric forcing on phase-locking to sinusoidal forcing is shown in figure 4; the state function $x(t)$ is shown for three values of parametric forcing amplitude, chosen to be fractions of the steady-state average value of the oscillations of the control parameter generated by the self-tuning equation, $\bar{\mu} = \langle \mu_{\text{self-tuning active}}(t) \rangle$. An example of the decrease in total response to sinusoidal forcing and blunting of frequency sensitivity (the widening of the response curves $\mathcal{A}(\omega')$) as a result of parametric forcing is shown in figure 5.

4 Self-Tuning and Sinusoidal Additive Forcing

Spontaneous oscillations produced by a Hopf oscillator endowed with the self-tuning equation for the control parameter $\mu(t)$ are similar in steady state to oscillations produced by a Hopf oscillator with a constant value of μ chosen to be the steady-state average value of the self-tuning control parameter oscillations, $\mu_{\text{self-tuning inactive}} = \bar{\mu}$. The responses of a self-tuned Hopf oscillator and a Hopf oscillator with $\mu = \mu_{\text{self-tuning inactive}}$ to sinusoidal forcing are shown in figure 6. The phase-locked amplitude $\mathcal{A}(F)$ is shown for both oscillators as a function of the amplitude of sinusoidal forcing at the common frequency of spontaneous oscillation in figure 7; it is clear that the oscillators display approximately identical response power laws.

Without the control parameter self-tuning equation, a Hopf oscillator remains on the oscillatory side of the bifurcation during large-amplitude overstimulating forcing, so the recovery time $t_R = 0$.

5 Self-Tuning and Overstimulating Forcing

Consistent with experimental observations of the properties of spontaneous hair bundle oscillations immediately after the recovery from overstimulating forcing, we found that, following large-amplitude constant forcing, post-recovery oscillations of a Hopf oscillator endowed with control parameter self-tuning grew in amplitude, shrunk in frequency, and grew in the per-cycle ratio of the length of the interval above the zero point of x to the length of the cycle (this quantity has been used as an estimate of the open probability of mechanotransduction channels.) These changes in the properties of post-recovery oscillations are shown in figures 8, 9, and 10.

6 Parametric Forcing of τ

We applied parametric forcing to the parameter μ by modifying the self-tuning equation to the following:

$$\frac{\mathrm{d}\mu}{\mathrm{d}t} = -\frac{\mu}{\tau F_p} - \frac{\alpha}{1 + e^{-\gamma x}} + C \tag{3}$$

Where the parametric forcing function $F_p(t) = \Gamma(\Theta(t - t^p_{\text{on}}) - \Theta(t - t^p_{\text{off}}))$ for forcing amplitude Γ. According to equation 1, the recovery time $t_R(\tau)$ is a monotonically increasing function of time constant τ, so parametric forcing with amplitude $\Gamma \ll 1$ of the time constant during the interval following cessation of overstimulating constant additive forcing has the effect of reducing the recovery time so that recovery is almost instantaneous. Such parametric forcing also causes the amplitude of spontaneous oscillations to decrease (the steady-state time average amplitude $\langle A \rangle \approx \sqrt{-\tau(C - (\alpha/2))/b'}$) and frequency to increase ($\langle \dot{\phi} \rangle \approx \omega - \tau b''(C - (\alpha/2))/b'$).

7 Denoising of Experimental Data

Experimental data were denoised using a first-order digital Butterworth filter with critical frequency 50 Hz calculated as cascaded biquadratic sections.

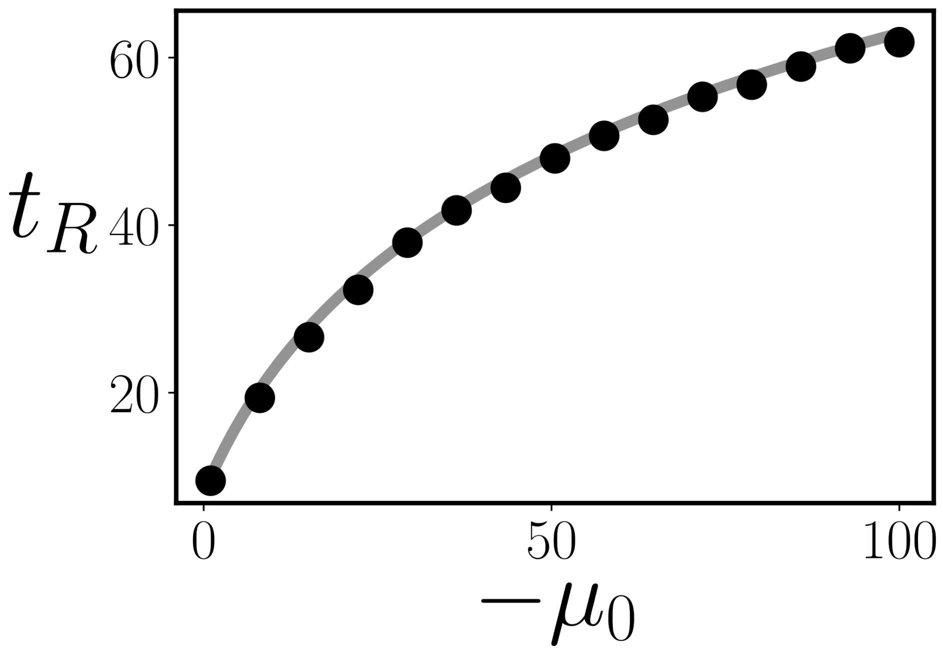

Figure 1: Duration $t_R(\mu_0)$ (black disks) preceding recovery of spontaneous oscillations from initial negative (quiescent) value of the control state parameter μ. The estimate defined by equation 1 is shown in gray.

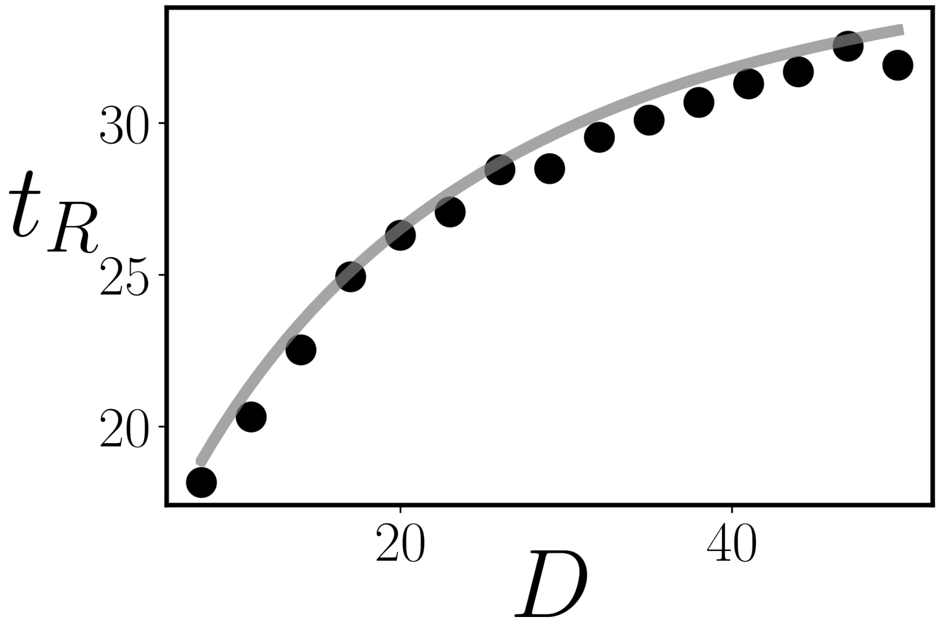

Figure 2: Duration $t_R(D)$ (black disks) preceding recovery of spontaneous oscillations from overstimulating constant forcing $F = [\Theta(t - t^a_{\mathrm{start}}) - \Theta(t - t^a_{\mathrm{stop}})]F$ with strength $F = 60$ and duration $D = t^a_{\mathrm{stop}} - t^a_{\mathrm{start}}$. The estimate defined by equation 2 is shown in gray.

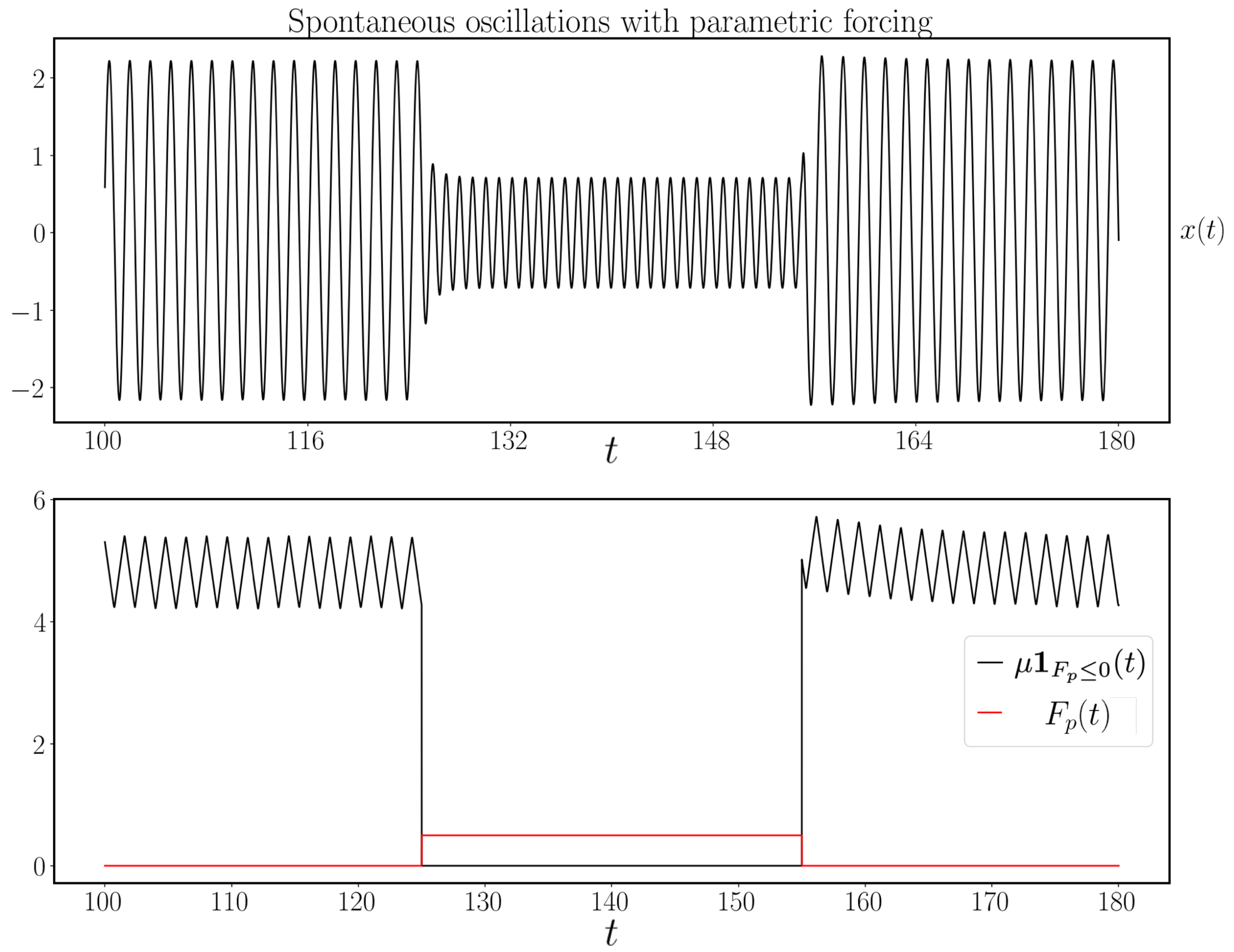

Figure 3: State function $x(t)$ (shown in black) for a Hopf oscillator with parametric forcing. Control parameter term $\mu(t)\mathbf{1}_{F_p \leq 0}(t)$ shown in lower panel (black curve) with rectangular parametric forcing function $F_p(t)$ (red curve.)

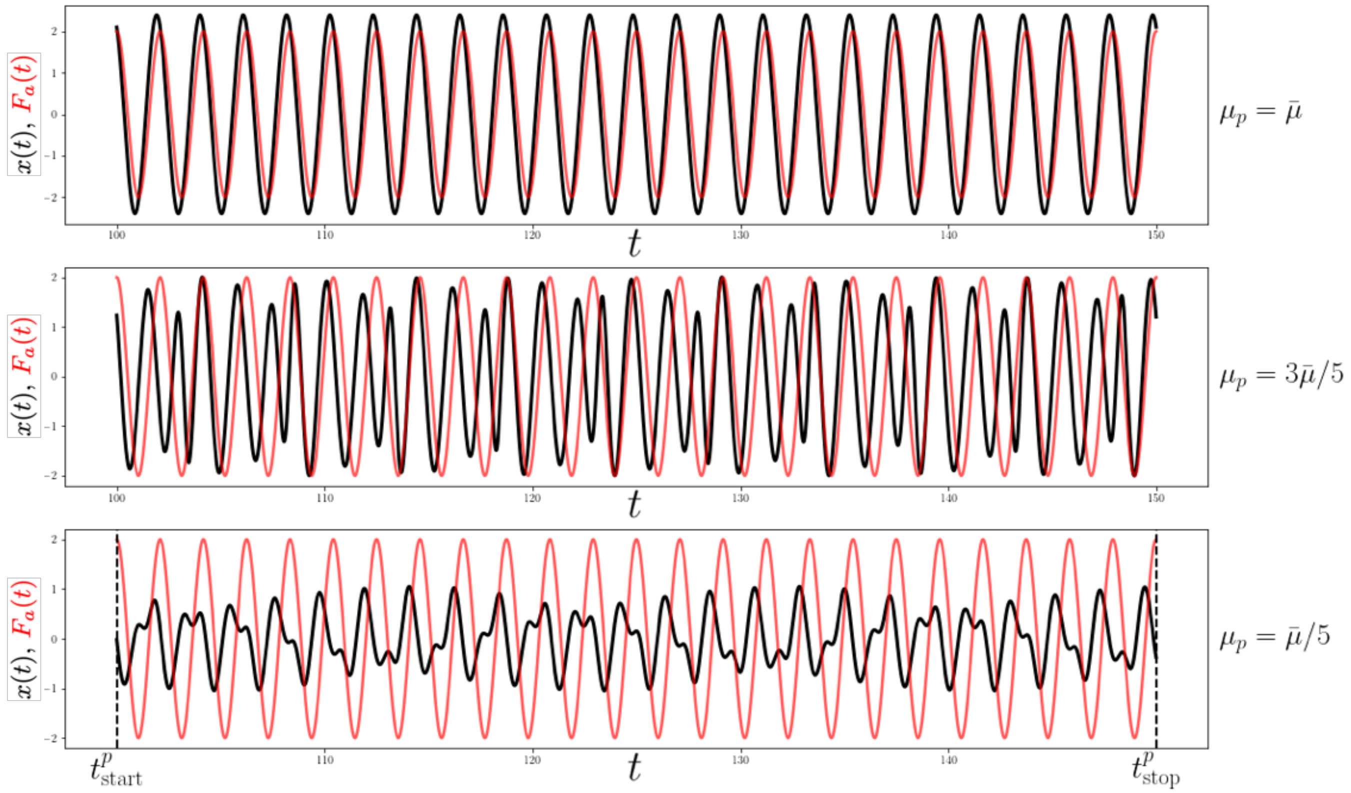

Figure 4: Effect of parametric forcing on phase-locking. Figure: response of state function $x(t)$ (shown in black) to identical additive forcing $F_a(t)$ (shown in red) for three values of parametric forcing amplitude μ_p.

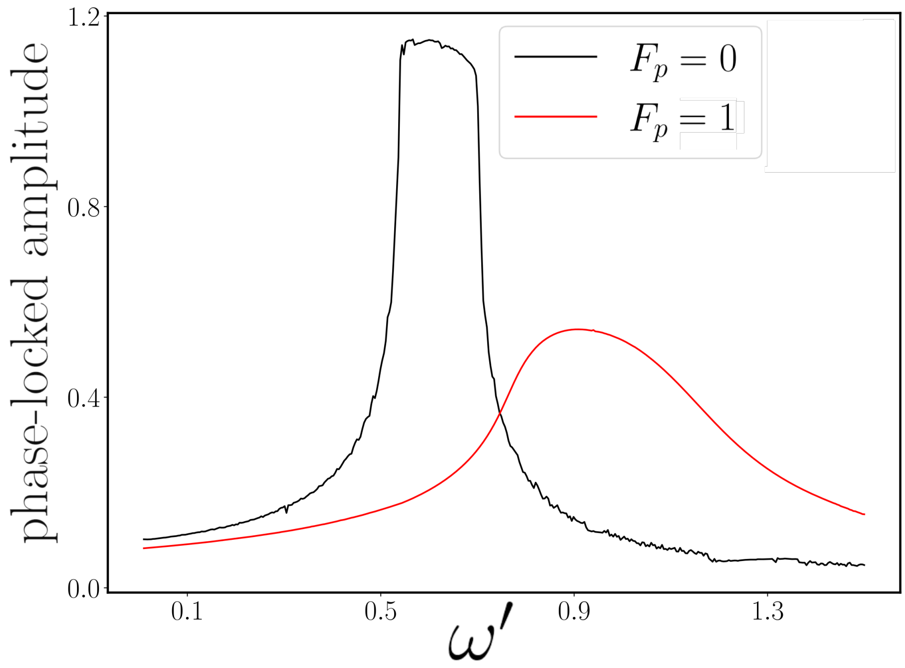

Figure 5: Parametric forcing causes the total phase-locked forcing response of a Hopf oscillator to sinusoidal forcing to decrease and widens the range of frequencies to which a Hopf oscillator is sensitive. Figure: normalized Fourier amplitudes $\mathcal{A}(\omega') = |\mathcal{F}[x(t)]|(\omega')$ representing 1:1 phase-locking to sinusoidal forcing $F_a = e^{i\omega' t}$ shown in the case of no parametric forcing and constant parametric forcing $F_p = \mu_p$ with amplitude $\mu_p = 1$.

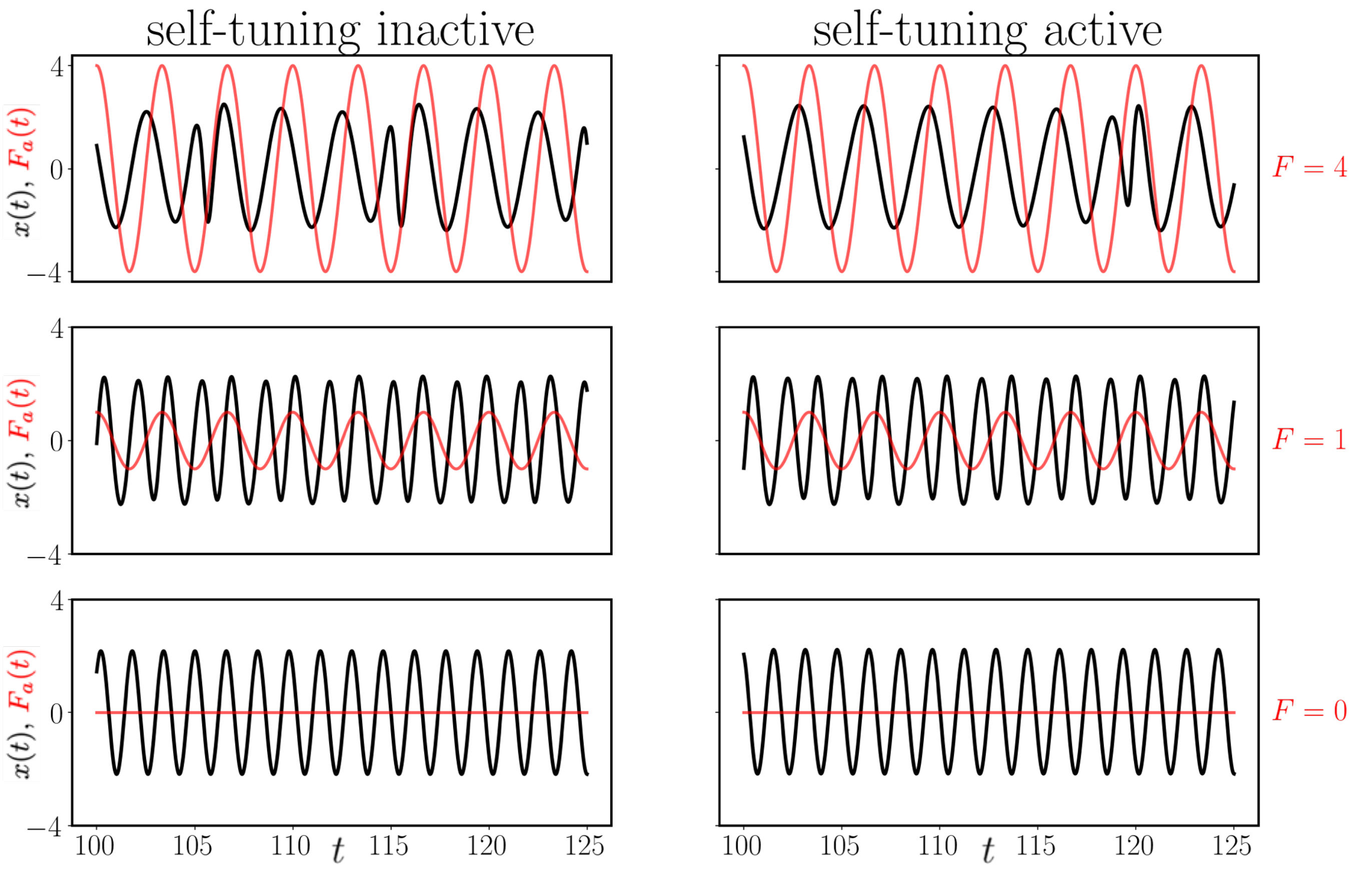

Figure 6: State functions $x(t) = \mathrm{Re}[z(t)]$ for (1) a Hopf oscillator with control parameter self-tuning feedback (left column) and (2) a Hopf oscillator with $\mathrm{d}\mu/\mathrm{d}t = 0$ and $\mu = \langle \bar{\mu}_{\mathrm{ss}} \rangle_{\mathrm{sample}}$, the average over additive forcing amplitudes of the steady-state average of the control parameter $\mu(t)$ subject to self-tuning (as in the right-hand column.) The amplitude of additive forcing $F_a = Fe^{i\omega' t}$ with $\omega' = 0.3$ (red solid lines) increases up from the bottom row. For both oscillators, the nonlinear parameter $\beta = -1 - 0.5i$ and the frequency parameter $\omega = 2\pi$.

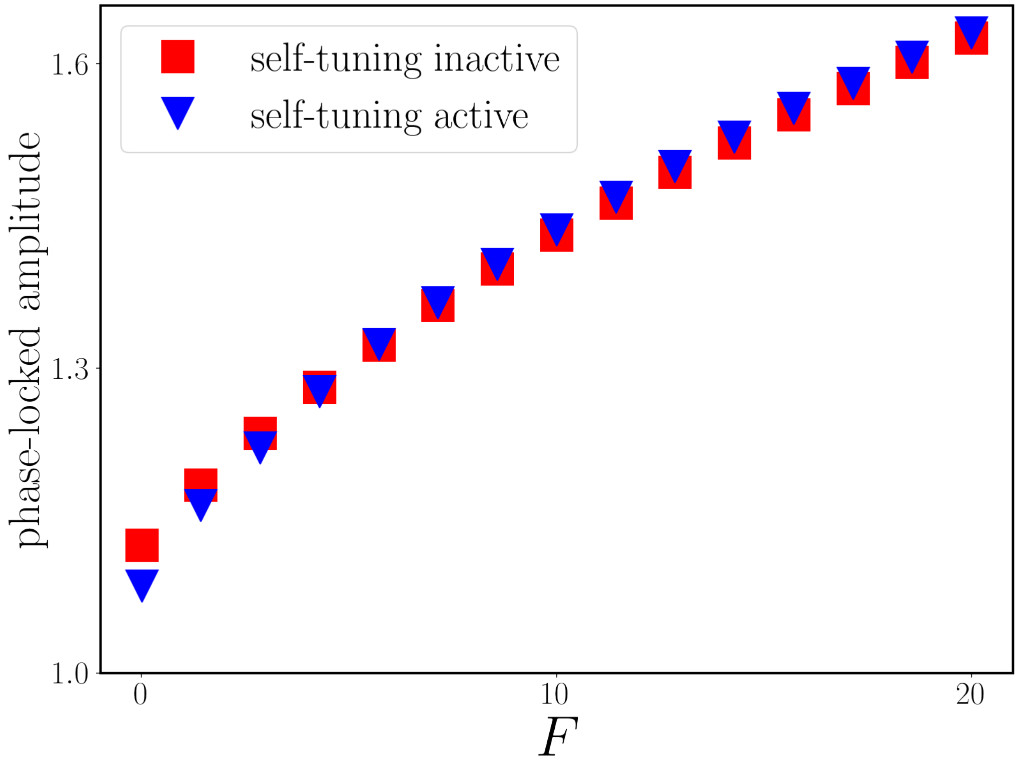

Figure 7: Phase-locked amplitude as a function of amplitude of additive sinusoidal forcing $F_a(t) = Fe^{i\omega' t}$ with $\omega' = \dot{\phi}$, the intrinsic frequency, for Hopf oscillators with and without control parameter self-tuning feedback.

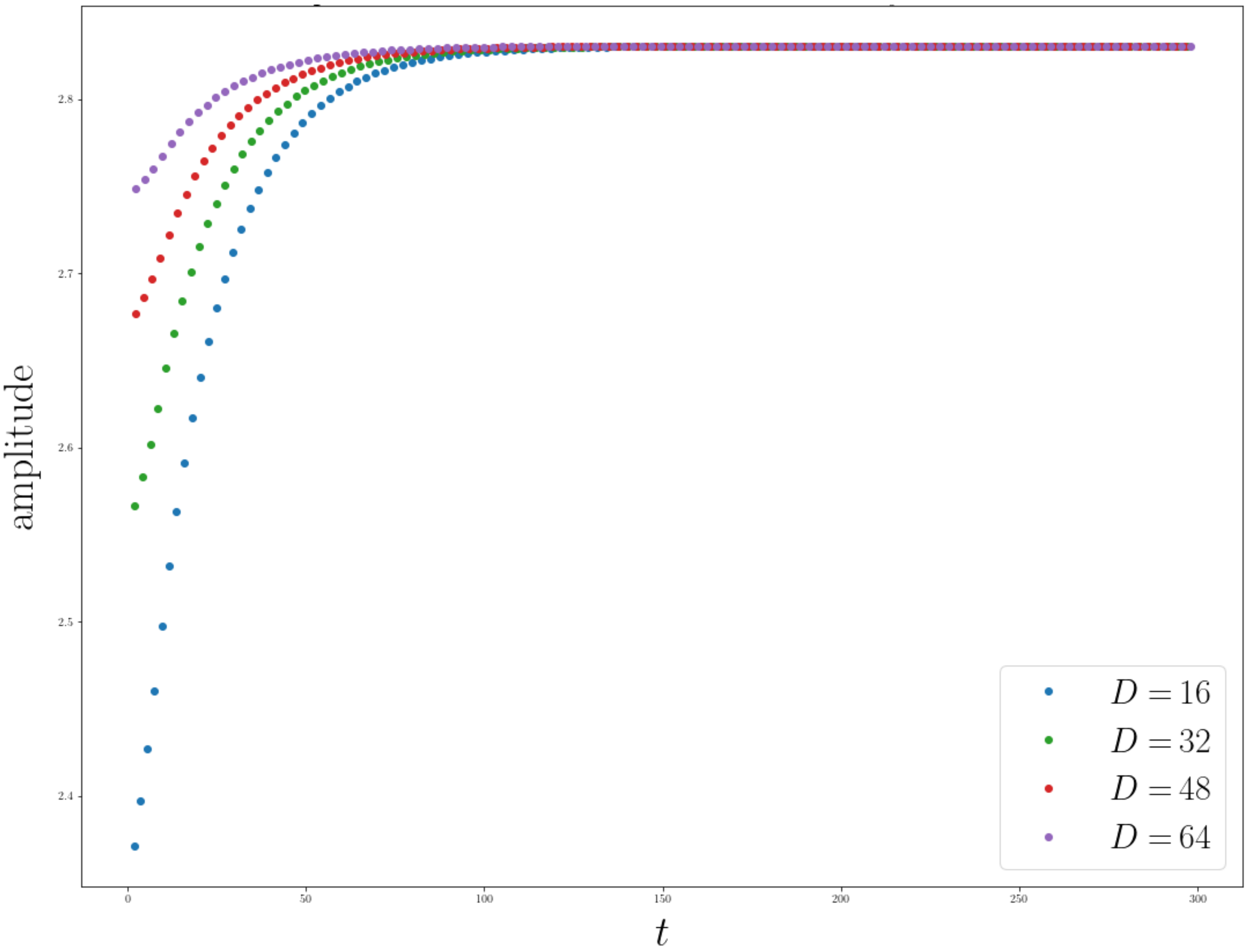

Figure 8: The effect of self-tuning on spontaneous oscillation amplitude following the cessation of overstimulating forcing for four forcing durations $D = t^a_{\text{start}} - t^a_{\text{stop}}$. Amplitude is measured as the moving mean of $(\langle x_{\text{above}}\rangle - \langle x_{\text{below}}\rangle)/2$ where $\langle x_{\text{above, below}}\rangle$ represent the empirical means within each cycle i of the values of x greater than and less than the value x_{midline} corresponding to the minimum of the Gaussian kernel density estimate of $x(t)$, respectively.

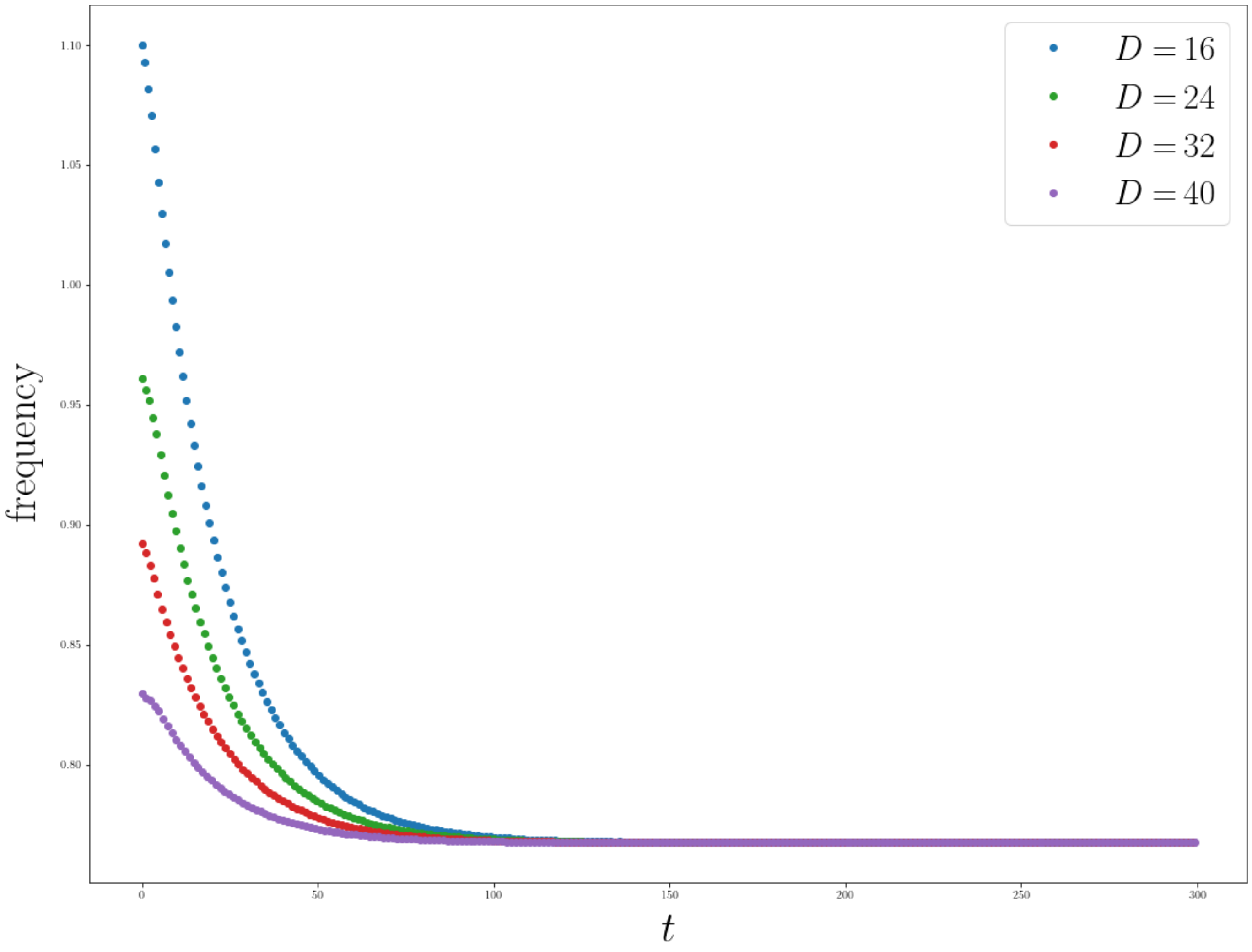

Figure 9: The effect of self-tuning on spontaneous oscillation frequency, measured as the moving mean of $f(t_i) = 1/T_i$ for period T_i of cycle i, following the cessation of overstimulating forcing.

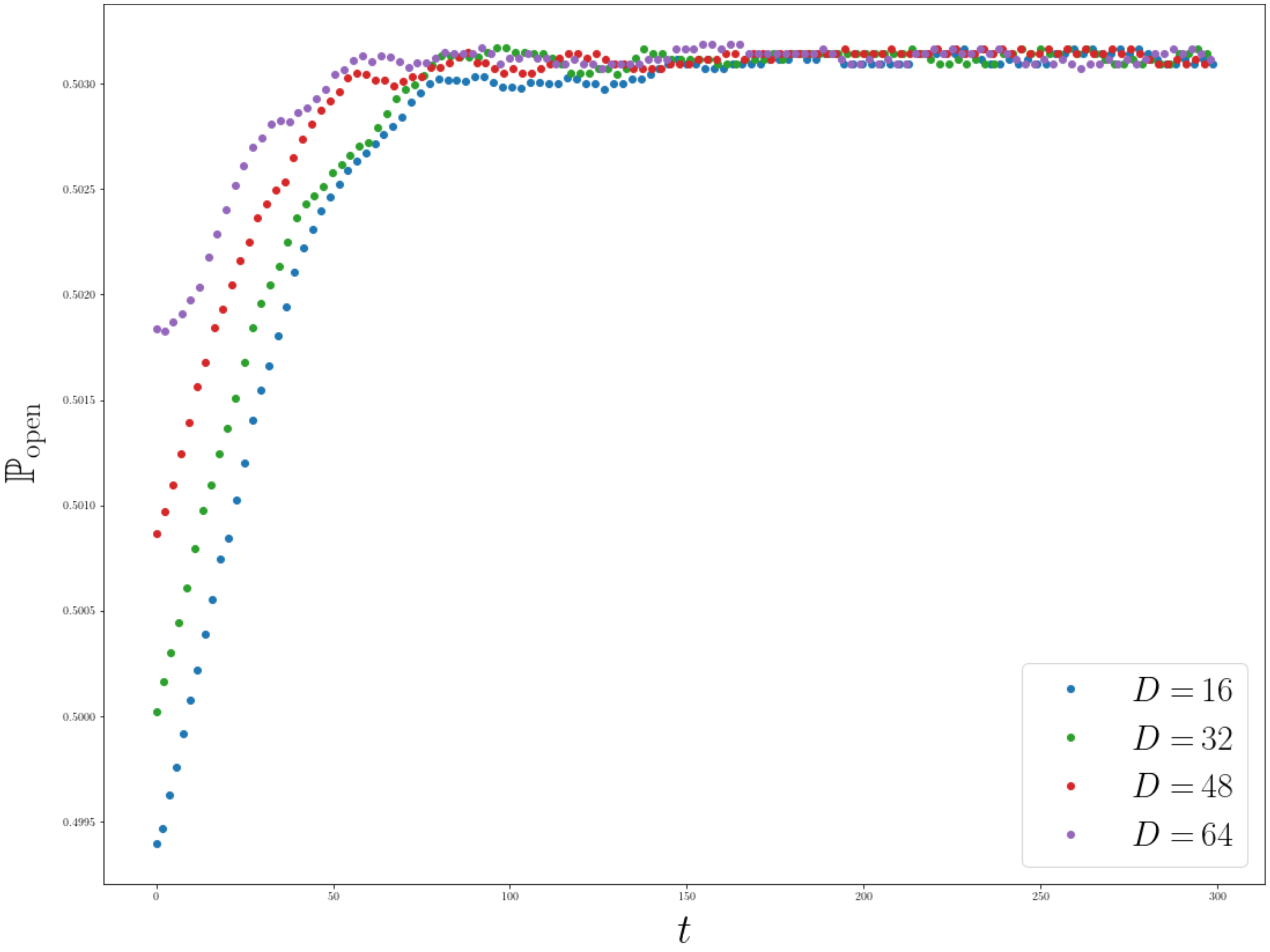

Figure 10: The effect of self-tuning on open probability, measured as the moving mean of the ratio within each cycle i of the number of samples for which all values of x are greater than x_{midline} to the total number of cycles in the cycle, following the cessation of overstimulating forcing.
