# Supplementary material for: Neural control and innate self-tuning of the hair cell’s active process
Source: Biophys J. 2024 Sep 6;123(20):3550–7. doi: 10.1016/j.bpj.2024.09.006 (PMC11494480; doi:10.1016/j.bpj.2024.09.006)
Supplement: Document S2. Article plus supporting material [file mmc2.pdf]

Biophysical Journal
Article

Biophysical Society

Neural control and innate self-tuning of the hair cell's active process

Charles Metzler-Winslow,[1,*] Martín A. Toderi,[1] and Dolores Bozovic[1,2]
[1]Department of Physics and Astronomy, University of California, Los Angeles, Los Angeles, California and [2]California NanoSystems Institute, University of California, Los Angeles, Los Angeles, California

ABSTRACT We propose a model for the feedback control processes that underlie the robustness and high sensitivity of mechanosensory hair cells. Our model encompasses self-tuning active processes intrinsic to these cells, which drive the amplification of mechanical stimuli by consuming metabolic energy, and a neural input process that protects these cells from damage caused by powerful stimuli. We explore the effects of these two feedback mechanisms on mechanical self-oscillations of the sense cells and their response to external forcing.

SIGNIFICANCE The sensory cells responsible for hearing rely on multiple feedback processes to fine-tune their response to sound. Intrinsic force feedback on hair bundles, the sensory organelles of these cells, underlies the extraordinary sensitivity of hearing and the spontaneous self-oscillations of hair bundles. Neural feedback further adjusts the sensitivity of hearing and the properties of hair bundle self-oscillations. We propose that intrinsic feedback can protect hair cells from damage by reducing their gain in response to loud sounds, and we show that both intrinsic feedback and neural feedback can be understood as processes that govern a parameter representing the propensity of the hair bundle to self-oscillate.

INTRODUCTION

Auditory systems of vertebrate organisms contain biological sensors that are capable of detecting extremely weak signals. At the quiet extreme of their dynamic range, hair cells of the inner ear reliably respond to sounds that deliver less power to their sensory organelles than background noise (1). The sensory organelle of the hair cell is the hair bundle; it comprises rows of stereocilia—long, narrow cylindrical extensions of the cell membrane—arranged in a staircase-like structure that protrudes from the surface of the cell (2,3). This array of stereocilia transduces mechanical deflections into ionic currents, which trigger the transmission of information about the auditory signal to the brainstem via the auditory nerve (4,5). At the loud extreme, hair cells detect sounds 12 orders of magnitude larger in intensity than the softest audible sounds, so the auditory system must balance sensitivity to weak stimuli with protection of the delicate machinery of hair cells from damage caused by powerful signals (6–9).

Submitted June 4, 2024, and accepted for publication September 3, 2024.
*Correspondence: metzler@ucla.edu
Editor: Richard Bertram.
https://doi.org/10.1016/j.bpj.2024.09.006
© 2024 The Author(s). Published by Elsevier Inc. on behalf of Biophysical Society.
This is an open access article under the CC BY license (http://creativecommons.org/licenses/by/4.0/).

Signatures of active mechanics

To achieve its extraordinary sensitivity, the auditory system must overcome significant viscous dissipation (1,10,11). Previous studies have revealed a rich network of cellular processes that is capable of counteracting damping by consuming metabolic energy (12–14). These energy-consuming processes explain the paradigmatic nonlinear features of hearing: amplification of soft sounds, sharp frequency discrimination, compression of the dynamic range of sound inputs, and self-oscillations (15–19).

The hearing system must also anticipate future stimuli by performing sensory adaptation to maximize sensitivity and prevent damage. While the centrality of active amplification is well established, the feedback processes that control the gain of the hearing system, thus protecting hair cells from damage caused by loud sounds, remain obscure. In this work, we present a model for hair cell dynamics that captures the effects of two such processes.

Efferent feedback

One process that has been shown to serve a protective role in hearing is efferent input. The axons of efferent neurons synapse onto the hair cell membrane, where postsynaptic

receptor activation triggers hyperpolarization. This flow of information from the brain to sensory cells modulates the sensitivity of hearing. Activation of efferent neurons reduces the hearing system's gain and blunts its frequency discrimination (for a review of efferent physiology, see (20)). When efferent neurons are severed, even sustained moderate-level stimuli permanently damage the hearing system (21).

Efferent neurons have also been shown to affect active cellular processes at the level of individual hair bundles. Activation of efferent neurons increases the threshold stimulus amplitude necessary to elicit a significant frequency-locked hair bundle response, rendering hair bundles less sensitive to applied stimuli. Furthermore, the innate active self-oscillations exhibited by hair bundles ex vivo (22) are inhibited by efferent activation.

Intrinsic hair cell feedback

Another source of robustness in the auditory system is the network of processes that suppress spontaneous hair bundle oscillations in response to large-amplitude stimulus forcing. Following the application of strong boxcar function (23) or sinusoidal (24) forcing ex vivo, hair cells exhibited an interval of quiescence after cessation of the stimulus before recovering oscillatory behavior, and the length of the quiescent interval increased with the duration of forcing. This effect was observed in biological preparations in which efferent neurons had been severed, so it indicates an intrinsic feedback process in hair cells that does not rely on neural feedback.

MATERIALS AND METHODS

We develop a simple theoretical model for the multifaceted feedback processes that regulate the response of auditory hair cells. Our nonlinear, continuous-time, deterministic model includes an innate, active self-tuning process described by a feedback differential equation governing a parameter that determines the propensity of the hair bundle to self-oscillate. We represent efferent input as parametric forcing that controls this parameter. Although our model is simple and requires few assumptions, it captures many intricate properties of the feedback phenomena that shape the response of auditory hair cells.

Innate spontaneous oscillations, amplification, dynamic range compression, and sharp frequency response are distinctive features of a dynamical system operating in the vicinity of a Hopf bifurcation. These bifurcations, which mark qualitative changes from quiescence to self-oscillation, have been identified in detailed biophysical models for hair bundle motion (2,14,25). The paradigmatic attributes of hair bundle motion are captured parsimoniously by a differential equation that is universal around any equilibrium that undergoes a Hopf bifurcation (26,27). In the most simple choice of coordinates, which can be attained via transformations dictated by normal form theory, this equation is called the Stuart-Landau equation:

$$\frac{\mathrm{d}z}{\mathrm{d}t} = (\mu_c + \mu)z + i\omega z + \beta|z|^2 z. \quad (1)$$

The complex variable $z(t) = x(t) + iy(t)$ represents a state function with a fixed point $z = 0$. The real part $x(t)$ represents the instantaneous position of the hair bundle along the line spanned by its height gradient. The control parameter μ represents the displacement from the supercritical Hopf bifurcation point. A transition in the stability of the fixed point $z = 0$ and the geometry of the orbits of Eq. 1 occurs across the bifurcation point μ_c. For values of the control parameter below the critical threshold value, $\mu < -\mu_c$, solutions to Eq. 1 decay to a stable node at $z = 0$. For values above the critical value, $\mu > -\mu_c$, the fixed point $z = 0$ loses stability, and solutions approach a stable limit cycle. The parameter ω represents the angular frequency of limit cycle oscillation approached as $\mu \to -\mu_c^+$, and the parameter $\beta = b' + ib''$ determines the strength of the nonlinearity. The real part b' governs the rate of growth of limit cycle oscillation amplitude $A = |z|$ as a function of the control parameter according to the amplitude equation:

$$A = \sqrt{-\frac{(\mu_c + \mu)}{b'}}. \quad (2)$$

The imaginary part b'' dictates the degree of coupling between the amplitude A and the angular frequency $\dot{\phi} = \mathrm{d}_t \arg(z)$ according to the frequency equation:

$$\dot{\phi} = \omega + b'' A^2. \quad (3)$$

When the amplitude and frequency are independent, $b'' = 0$, the oscillator is termed isochronous.

Response to external forcing

We represent stimulus using an additive forcing function $F_a(t)$. Including this external stimulus, the governing equation becomes

$$\frac{\mathrm{d}z}{\mathrm{d}t} = \left(\mu_c + \mu + i\omega + \beta|z|^2\right)z + F_a. \quad (4)$$

For a pure tone stimulus applied at the characteristic frequency of the oscillator $F_a(t) = Fe^{i\omega t}$, the response amplitude at the bifurcation point is given by a one-third power law:

$$A = -b'^{-1/3} F^{1/3}. \quad (5)$$

Hence, the gain characterizing sensitivity diverges in the $F \to 0$ limit, $\mathrm{d}A/\mathrm{d}F = b'^{-1/3} F^{-2/3}/3$. For forcing frequencies sufficiently far from ω, A is linear in F. A one-third power law for the resonant response has been observed ex vivo for external forcing amplitudes spanning much of the total physiologically relevant range (28).

Considering stimulus frequencies on each side of the characteristic frequency, the width at half-maximum of the amplitude function $A(\omega', F)$ decreases with decreasing stimulus amplitude:

$$\Delta\omega'_{A_{\max}/2} \propto F^{2/3}. \quad (6)$$

The width of response has been experimentally shown to increase with the strength of forcing. Hence, small-amplitude stimuli induce narrowly tuned high-gain responses around the characteristic frequency, while large-amplitude stimuli induce broadly tuned low-gain responses (26). These response properties differ sharply from those of passive resonating systems such as the driven damped harmonic oscillator, for which the response width and gain are both independent of forcing amplitude at all forcing frequencies.

Critical self-tuning

We include a self-tuning equation that automatically maintains the system in the proximity of the critical point $\mu = -\mu_c$. Since the one-third power

law for the response amplitude holds at the critical point, while the response is linear away from the critical point for sufficiently small forcing amplitudes, such a feedback control process would maintain the high sensitivity of hair cells to soft sounds and ensure a wide dynamic range (26). A system that self-tunes to the vicinity of a Hopf bifurcation, like the one we study here, is termed a critical oscillator (8,29–32).

In anamniote species and some amniotes, the intrinsic cellular processes underlying sensitivity and spontaneous oscillation have been localized to the hair bundle. These active mechanisms involve the movement of myosin motors and changes to the probability that transduction channels will close (25). Earlier work has indicated that Ca^{2+} ions, which flow into stereocilia as part of the hair cell transduction current, serve as a crucial regulator of active hair cell mechanisms. The effects of Ca^{2+} on these active mechanisms can be broadly classified as negative feedback: Ca^{2+} reduces the force exerted by myosin motors and makes transduction channels more likely to close (25,33).

We note that a different set of active mechanisms are thought to underlie sensitivity in the hair cells of mammalian organisms (34,35). We emphasize that the abstract representation of sensitivity in the universal framework based on the Stuart-Landau equation is independent of the particular active mechanisms underlying sensitivity.

We interpret the control parameter as a measure of the degree of intrinsic hair cell activity (see Eq. 2). Assuming the degree of activity is negatively affected by Ca^{2+}, we employ a specific self-tuning equation introduced in (8):

$$\frac{d\mu}{dt} = -\frac{\mu}{\tau} - \frac{\alpha}{1 + e^{-\gamma x}}. \tag{7}$$

The first term on the right-hand side of Eq. 7 produces relaxation to the value of the second term with a time constant τ. The second term represents the effect of Ca^{2+} influx on the control parameter. This term follows from a simplified model of transduction channels and tip links, which are elastic protein strands that connect the transduction channel complex in each stereocilium to the side of the tallest neighboring stereocilium; each tip link is modeled as a Hookean spring connected to a transduction channel that can only occupy either a closed or open state. A canonical ensemble of such pairs of tip links and channels yields an open state probability P_o that is a logistic function of the bundle displacement x. Thus, the middle term $\alpha P_o(\gamma, x)$ represents the effect of the total influx of Ca^{2+} on the degree of hair cell activity, where α represents the strength of the effect and is proportional to the constant influx through a single open channel multiplied by the total number of channels. At constant temperature, the parameter γ is proportional to the force associated with the transition from the closed to open channel state.

(36) argues that, for a large collection of systems, a control feedback law of the form $\dot{\mu} = f(A) - g(\mu)$ maintains μ near the critical point $-\mu_c$ if f and g satisfy the conditions that 1) f is a decreasing function of amplitude A, 2) g is an increasing function, and 3) there exists a value A_0 for which $\dot{\mu} = 0$ at the critical point, $f(A_0) = g(-\mu_c)$. Eq. 7 satisfies those conditions and, indeed, dictates that the steady-state time average $\langle\mu\rangle$ grows to approximately $-\alpha\tau/2$, a value near the critical point $-\mu_c$, in the absence of forcing when $\mu_c < \tau\alpha$.

Parametric forcing

We introduce a parametric forcing function $F_p(t)$ to account for the effects of efferent input on hair bundle mechanics. We note that parametric forcing has been applied previously to the study of the auditory system; it provides a simple theoretical framework for describing a complex network of feedback processes (37).

While the biophysical mechanisms that produce the effects of efferent input on hair bundle mechanics remain unknown, prior work has suggested that the effects are mediated by calcium ions. The influx of Ca^{2+} into stereocilia is controlled by its transmembrane concentration gradient and the transmembrane gradient of electric potential. Since efferent input hyperpolarizes hair cells, it increases the force driving Ca^{2+} influx through the transduction channels. Hence, by modulating the influx of Ca^{2+}, efferent activation exerts an effect on hair bundle mechanics via the active mechanisms internal to hair cells.

When strong boxcar forcing was combined with efferent input, efferent feedback supplanted intrinsic feedback. Following cessation of large-amplitude constant mechanical stimulus, efferent nerve fiber activation caused a dramatic decrease in the length of the quiescent interval (the recovery of oscillations was very rapid when and only when efferent nerve fibers were activated) (38). Inspired by this experimental observation, we hypothesize that strong, persistent efferent synaptic activity, which we model using a constant parametric forcing term, overwhelms the internal cell dynamics responsible for the transition to quiescence and recovery of oscillations. We modify the governing equation so that F_p replaces the control parameter μ:

$$\frac{dz}{dt} = \left(\mu_c + \mu 1_{F_p = 0} + F_p\right)z + i\omega z + \beta|z|^2 z + F_a. \tag{8}$$

Here, 1 denotes the indicator function, and we emphasize that the first term in Eq. 8 reflects the hypothesis that efferent activation supplants intrinsic feedback: the parametric forcing function $F_p(t)$ replaces $\mu(t)$ when $|F_p| > 0$.

Experimental methods

Sacculi from the North American bullfrog (*Rana catesbeiana*) were dissected maintaining their full physiological integrity (39). Hair cells were imaged ex vivo during spontaneous oscillations of the hair bundle and electrical stimulation of the eighth cranial nerve (38). These perturbations served to trigger or modulate mechanical motions of the hair bundle. Optical imaging was then used to detect fluctuations in the light intensity of pixels, serving to determine the hair bundle position over time at the upper limit transverse sectioning plane.

Frogs of either gender were anesthetized (pentobarbital: 150 mg/kg), pithed, and decapitated following protocols approved by the University of California, Los Angeles Chancellor's Animals Research Committee. Sacculi were excised from the inner ears of the animals and placed in oxygenated artificial perilymph solution (in mM as follows: 110 Na^+, 2 K^+, 1.5 Ca^{2+}, 113 Cl^-, 3 D-(+)-glucose, 1 Na^+ pyruvate, 1 creatine, 5 HEPES). The epithelium was mounted in a two-compartment chamber, emulating the fluid partitioning of the in vivo physiological conditions. In this arrangement, apical surfaces were bathed in artificial endolymph (in mM as follows: 2 Na^+, 118 K^+, 0.25 Ca^{2+}, 118 Cl^-, 3 D-(+)-glucose, 5 HEPES) and basolateral membranes in perilymph (40). In order to allow direct mechanical access to the hair bundles, the otolithic membrane was carefully removed from the epithelium after an 8 min enzymatic dissociation with 15 g/mL collagenase IV (Sigma-Aldrich, St. Louis, MO, USA).

Recordings were performed using an upright optical microscope (Olympus BX51WI, Tokyo, Japan) with a water-immersion objective (Olympus LUMPlanFL *N* 60×, NA: 1.00) mounted on an optical table (Technical Manufacturing, Durham, CT, USA). The setup was constructed inside an acoustically isolated chamber (Industrial Acoustics, Naperville, IL, USA) to avoid introducing external perturbations to the highly sensitive hair cells. 16 bit TIFF images at a resolution of 108.3 nm/px were recorded with a high-speed camera (ORCA-Flash4.0 CMOS) at 1000 frames per second. Motion of the hair bundles was tracked using custom-made MATLAB scripts. Specifically, the centroid of the hair bundle position was determined from the two-dimensional light intensity profile in each frame of the recording. Plots of bundle position over time then provided traces of its motion.

Electrical signals were applied to the eighth cranial nerve, producing alterations of the spontaneous oscillatory pattern of the hair cells. Hair

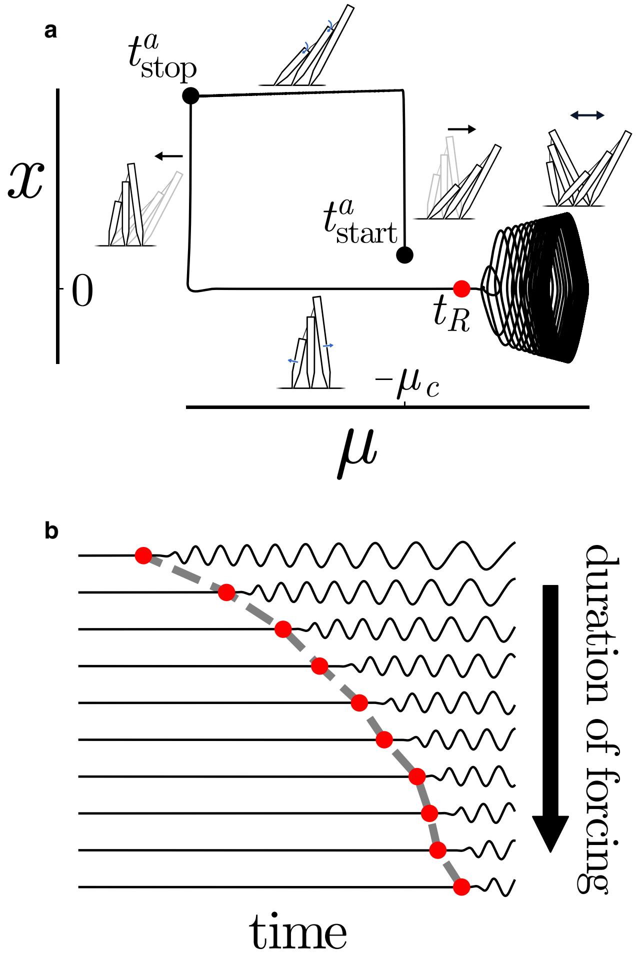

FIGURE 1 Self-tuning mediates the transition to quiescence and recovery of oscillations in response to strong constant external stimulus. (*a*) A large additive boxcar force $F_a = [\Theta(t - t^a_{start}) - \Theta(t - t^a_{stop})]F$ displaces the hair bundle to a maximal position. The hair bundle position then gradually relaxes. The deflection triggers feedback on the control parameter μ, which decreases during external forcing and reaches a value on the quiescent side of the critical point, $\mu(t^a_{stop}) < -\mu_c$. After forcing stops, the bundle returns to its resting position and remains quiescent, while the self-tuning mechanism restores μ to its steady-state value. Oscillations recover at time t_R after μ crosses the critical point, and the amplitude and frequency of oscillation approach their respective steady-state values asymptotically. Black arrows represent displacements, and blue arrows represent ion fluxes (specifically, *blue arrows* denote the transduction current in the upper schematic and clearance via extrusion pumps in the lower schematic). (*b*) The duration preceding recovery of spontaneous oscillations following large constant additive forcing is an increasing function of the duration of forcing. The position $x(t > t^a_{stop})$ (*black curves*) is shown for a collection of forcing durations from $t^a_{stop} - t^a_{start} = 40$ (*top*) to 97.5 (*bottom*). Recovery times t_R are shown in red. For these simulations, the amplitude $F = 1000$ and the time constant $\tau = 35$.

bundles oscillated with a noticeably higher frequency during activation of the efferent neurons than without stimulation. Efferent stimulation was performed with a bipolar suction electrode (A-M Systems, Sequim, WA, USA). The saccular nerve was pulled into a 0.5-mm-diameter silicon tube filled with perilymph, which was electrically connected to the positive terminal of the electrode, while the reference terminal was immersed in the perilymph compartment of the chamber. A linear stimulus isolator (World Precision Instruments A395, Sarasota, FL, USA) provided current to the suction electrode, and stimulus protocols were sent to the isolator via LabView (National Instruments, Austin, TX, USA). Constant step shaped stimuli were applied with current intensities of 50, 100, 150, and 200 μA for 1 s, preceded and followed by 2 s of no stimulation. Altered bundle oscillations were observed immediately after the onset of the stimulus and ceased upon its termination. Tracking the hair bundle movement allowed us to confirm proper activation of the efferent pathway and the induced changes in hair bundle motility observed in prior literature (22).

RESULTS

We suggest that the suppression and recovery of oscillations following strong constant mechanical stimulation are explained by the self-tuning dynamics of the control parameter $\mu(t)$: during stimulation, the influx term in Eq. 7 drives μ below the critical point, $\mu < -\mu_c$, where the system is quiescent. After stimulation, the control parameter returns to the oscillatory side of the critical point, $\mu > -\mu_c$, and settles into steady-state oscillation as shown in Fig. 1 *a*. This description is consistent with the observation that the length of the induced quiescent interval preceding the recovery of oscillations is an increasing function of stimulus duration: during stimulation, the displacement of $\mu(t)$ from the critical point increases with time. The recovery time is shown as a function of forcing duration in Fig. 1 *b*.

We find that parametric forcing explains the effects of efferent activity on spontaneous oscillations. For strong, persistent efferent activity, we introduce the parametric forcing function $F_p = (\mu_p - \mu_c)[\Theta(t - t^p_{start}) - \Theta(t - t^p_{stop})]$, where Θ denotes the Heaviside step function and the forcing amplitude $\mu_p < \mu_c + \langle\mu\rangle|_{F_p=0}$ (see Table 1). Consistent with experimental observations (22), this forcing reduces the amplitude and increases the frequency of spontaneous oscillation (Fig. 2). These effects are consistent with the effects of hyperpolarization of the hair cell membrane via the voltage clamp method, reinforcing the view that the central consequence of efferent activity is hyperpolarization of the cell soma (41,42).

Hair bundles have been shown to phase lock to sinusoidal forcing within a range of forcing frequencies around their frequency of spontaneous oscillation. The region of amplitudes and frequencies over which phase locking occurs is called the Arnold tongue. Consistent with the experimental observation that the response amplitudes over a hair bundle Arnold tongue were vastly reduced by efferent activity (22), constant parametric forcing decreased the phase-locked response amplitudes over the critical oscillator's Arnold tongue. This can be seen in Fig. 3, *a–c*, which depicts the forcing frequency component of the oscillator's magnitude spectrum. The insets of Fig. 3, *a–c*, depict the degree of phase synchronization over the Arnold tongue. The inset Arnold tongues widened in response to increasing parametric forcing strength, which we define as $S = 1/\mu_p$, so

TABLE 1 Parameter values unless otherwise specified

Parameter	Definition	Value
μ_c	bifurcation point	20
ω	characteristic angular frequency	2π
β	strength of nonlinearity	$-1-i/2$
τ	relaxation time	10
γ	sensitivity of self-tuning	10
α	strength of self-tuning	3

parametric forcing blunted the oscillator's frequency selectivity. The rate of widening is shown for a particular amplitude of external forcing in Fig. 3 *f*. We note that the reduction in the total phase-locked response as a result of parametric forcing—as illustrated in Fig. 3 *d*—is more dramatic for soft sounds than loud sounds.

The phase-locked response amplitude for off-resonance external forcing is shown as a function of parametric forcing amplitude in Fig. 3 *e*. On the quiescent side of the bifurcation point, the position function $x(t)$ is completely entrained to the external forcing. Close to the bifurcation on the oscillatory side, the oscillator remains entrained, and the intrinsic energy quantified by μ_p contributes to the phase-locked response amplitude. When the parametric forcing amplitude exceeds a threshold value, harmonics of the forcing frequency emerge in the oscillator response, and the phase-locked response amplitude decreases. For sufficiently large values of μ_p, the limit cycle frequency mode dominates.

Our model reproduces the experimental observation (38) that efferent activity can eliminate the transition to and recovery from quiescence induced by strong constant mechanical stimulation (Fig. 4). Oscillations recover immediately following large-amplitude constant external forcing when, and only when, parametric forcing is applied. In contrast, when parametric forcing is applied concurrently with external forcing, it does not affect the length of the quiescent interval since the control parameter $\mu(t)$ and the parametric forcing function $F_p(t)$ are independent.

DISCUSSION

We developed a theoretical model for the effects of internal self-tuning feedback and efferent input on active hair bundle dynamics. The model captures modulation of the amplitude and frequency of spontaneous oscillations by efferent activity and the reduced sensitivity of a hair cell to sinusoidal mechanical stimulus. We note that this reduction in sensitivity might reflect the protective role of efferent input. The model also reproduces the interaction between self-tuning and efferent activity in the context of the response to large-amplitude forcing. We emphasize that the elements of this model are generic and simple: a dynamical system defined by a universal equation for motion near a transition between quiescent and self-oscillatory states, dynamic feedback that automatically poises that

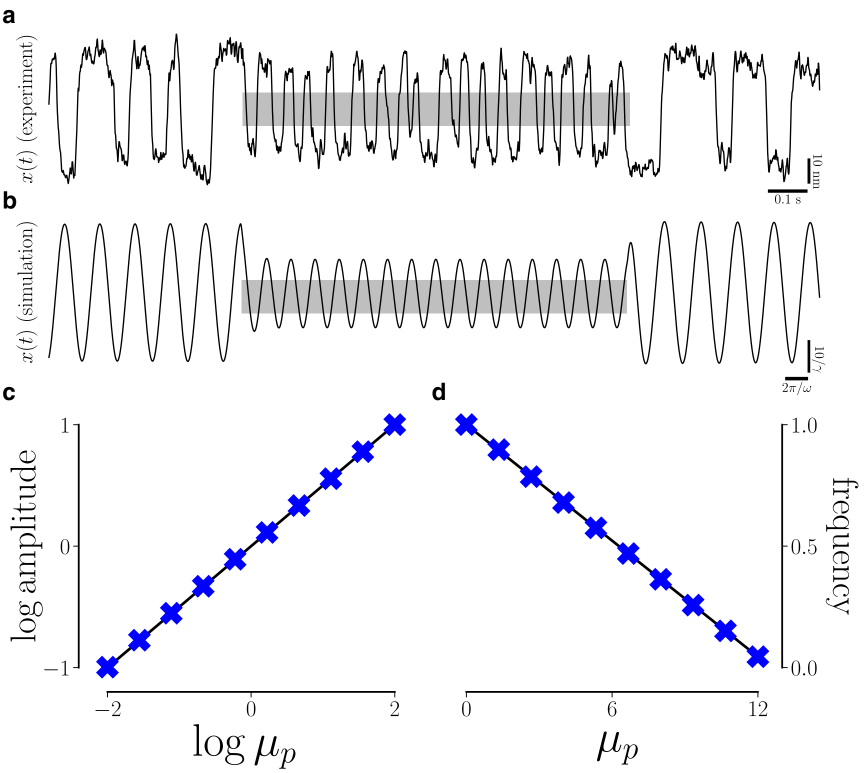

FIGURE 2 Parametric forcing reduces the amplitude and increases the frequency of spontaneous oscillations. (*a*) The hair bundle position $x(t)$ during spontaneous oscillations obtained via microscopic imaging. Efferent neurons were electrically activated during the interval spanned by the gray rectangle. (*b*) The position $x(t)$ obtained via numerical simulation with rectangular parametric forcing $F_p = [\Theta(t - t^p_{\text{start}}) - \Theta(t - t^p_{\text{stop}})](1-\mu_c)$. During the interval shown in gray, $[t^p_{\text{start}}, t^p_{\text{stop}}]$, the effects of parametric forcing match the experimentally observed effects of efferent activation on spontaneous oscillations. (*c* and *d*) Predicted steady-state amplitude of oscillation $\langle A \rangle = \sqrt{-\mu_p/b'}$ and frequency $\langle \dot{\phi} \rangle = \omega_0 - (b''/b')\mu_p$ (*black solid curves*). The empirical average was taken over 50 steady-state cycles of amplitude function $A(t) = |z(t)|$ and frequency function $\dot{\phi}(t) = d_t \arg(z)$ (*symbol x*) obtained via numerical simulation with $F_p = \mu_p - \mu_c$ for the entire stimulation duration.

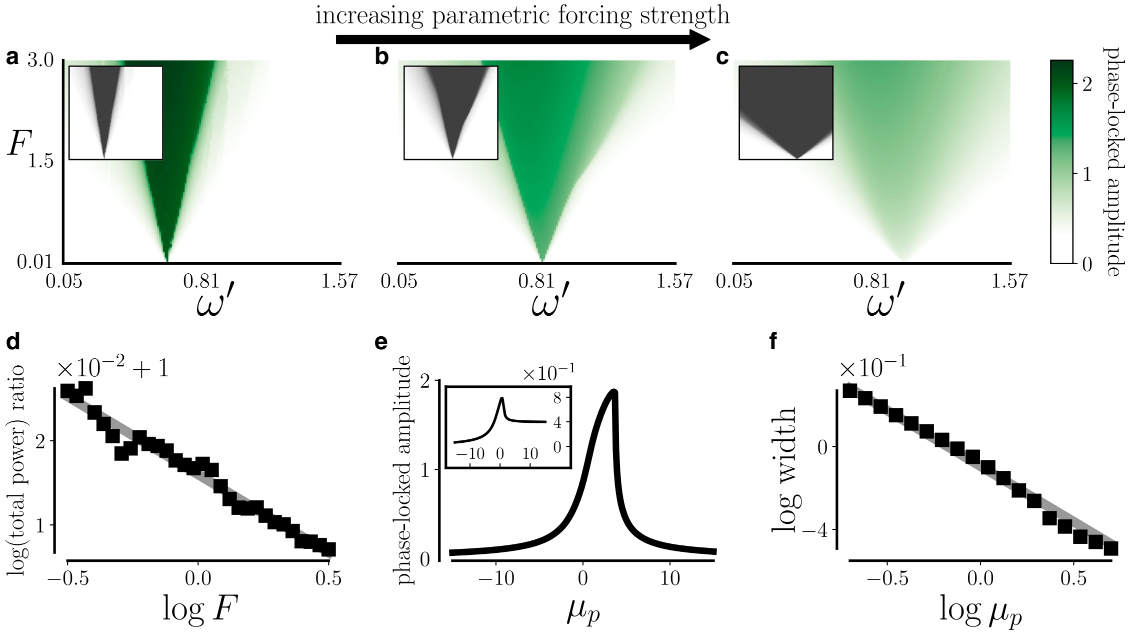

FIGURE 3 Constant parametric forcing $F_p = \mu_p - \mu_c$ changes the nonlinear response of a Hopf oscillator to sinusoidal additive forcing $F_a(t) = Fe^{i\omega' t}$. (*a–c*) The region of significant phase-locked amplitudes $\mathcal{A}(\omega') = |\mathcal{F}(z(t))|(\omega')$ shifts toward larger forcing amplitudes and spreads out across forcing frequencies with increasing parametric forcing strength $S = \mu_p^{-1}$. (*a*) represents the case of no parametric forcing, (*b*) parametric forcing with strength $S = 2/5$, and (*c*) parametric forcing with $S = 32/5$. (*a–c*) Insets: the vector strength $\mathcal{V}$, an estimate of the coherence function between $x(t)$ and $F_a(t)$, is shown in gray (frequency and amplitude axes identical between *images* and *insets*). $\mathcal{V} = \left|\langle e^{i(\phi-\phi')}\rangle\right|$, where $\phi(t)$ represents the angular displacement of z and $\phi' = \omega' t$. (*d*) Ratio R of total logarithmic powers, $R = \log_{10}\Sigma_{\omega'} P_{\text{inactive}}(\omega')/\log_{10}\Sigma_{\omega'} P_{\text{moderate}}(\omega')$, where P_{inactive} and P_{moderate} denote power spectral densities of $x(t)$ estimated using Bartlett's method with $\mu_p = 0$ and $\mu_p = 5/2$, respectively. In this case, $R \approx 1 + \log_{10} F^{-0.019}$. (*e*) Phase-locked amplitudes to forcing with $F = 1$ and $\omega' = 8\pi/5$ are shown as a function of the amplitude of parametric forcing of a Hopf oscillator in the isochronous and nonisochronous case, $\beta = -1$ (*inset*) and $\beta = -1 - i/2$ (*image*) respectively. We note that for both the isochronous and nonisochronous oscillators, the amplitude $\mathcal{A}(\mu_p)$ increases between the bifurcation point $\mu_p = 0$ and the point of maximal response, $\text{argmax}_{\mu_p}[\mathcal{A}] > 0$, so phase-locked responses are weaker closer to the bifurcation point on the oscillatory side. (*f*) Width at half-maximum $\Delta\omega'_{\mathcal{V}_{\max/2}}$ of the vector strength for forcing amplitude $F = 1.5$ as a function of parametric forcing amplitude μ_p. Here, $\Delta\omega'_{\mathcal{V}_{\max/2}} \approx -9.5 \times 10^{-2} + \mu_p^{-0.54}$.

system at the threshold of its transition, and parametric forcing. With these three elements, we recover the effects of signals flowing from the brain to hair cells in shaping active hair bundle mechanics.

Earlier theoretical and experimental works have examined various quantities that mediate a transition between the quiescent and oscillatory states of hair bundle motion. These quantities include the hair cell membrane potential, the stiffness of stereociliary pivots, the stiffness of an elastic load on the hair bundle, the extracellular concentration of Ca^{2+} near the tips of stereocilia, and the intracellular Ca^{2+} concentration. While each of these quantities constitutes a candidate for the control parameter, we note that the identity of the single control parameter μ remains uncertain (40–43).

Specific models of hair bundle mechanics include biophysical mechanisms for the regulation of the active network of intrinsic processes underlying spontaneous oscillations and the sensitivity of detection (25). The effects of Ca^{2+} concentration on the amplitude and frequency of spontaneous oscillations predicted by these models are consistent with our description of feedback on the control parameter μ. Hence, our findings are compatible with the view that the qualitative dynamic state of a hair bundle defined by the internal control parameter μ is affected by Ca^{2+}.

Although we focused on the impact of intrinsic and efferent feedback on hair bundle motion, our model aimed for generality, and we speculate that our model might provide theoretical insight into inhibitory feedback processes of sense cells in other systems. We suggest that parametric forcing of a quantity controlling gain can capture the key features of the modulation of cellular feedback processes that underlie sensitivity. Also, the self-tuning Eq. 7 contains elements that are likely applicable to many biological systems that contain self-regulation mechanisms mediated by ion concentrations: 1) relaxation dynamics and 2) influx through transduction channels.

Because recent experimental evidence indicates that efferent inputs to hair cells strongly influence their sensitivity and mechanics, we argue that theoretical models that aim to capture the full range of properties of the auditory sensory system should include efferent neural feedback.

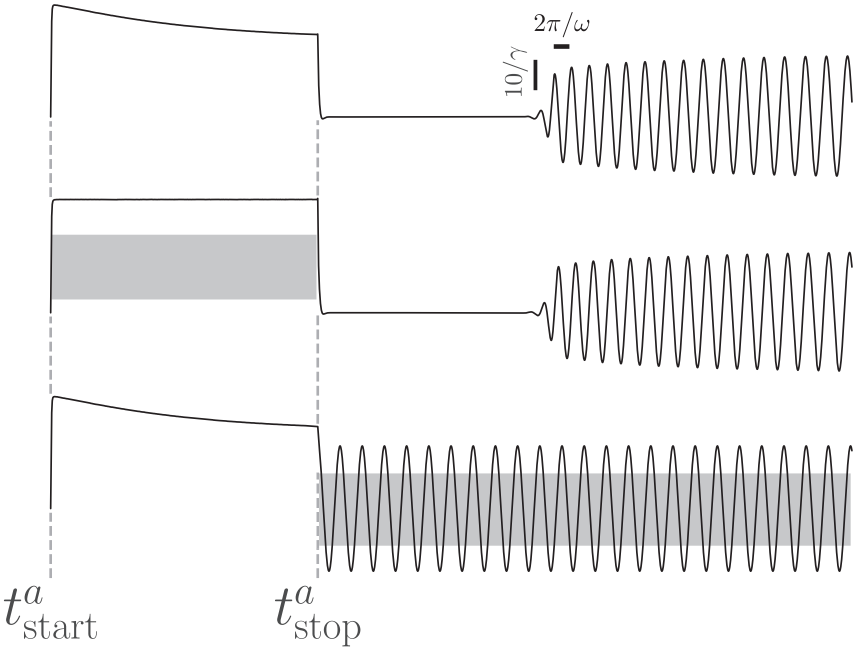

FIGURE 4 Parametric forcing causes the time before recovery of oscillations after large-amplitude forcing to vanish, and the application of parametric forcing during the interval following large-amplitude forcing is sufficient for the recovery of oscillations. Position $x(t)$ in response to overstimulating additive forcing $F_a = 50[\Theta(t - t^a_{\text{start}}) - \Theta(t - t^a_{\text{stop}})]$ with no parametric forcing (*top*) parametric forcing $F_p(t) = (5 - \mu_c)[\Theta(t - t^p_{\text{start}}) - \Theta(t - t^p_{\text{stop}})]$ applied only during overstimulating forcing (*middle*, interval $[t^p_{\text{start}}, t^p_{\text{stop}}] = [t^a_{\text{start}}, t^a_{\text{stop}}]$ shown in *gray*) and parametric forcing $F_p(t)$ applied after the cessation of overstimulating forcing until the end of the simulation at $t = t_{\text{end}}$ (*bottom*, interval $[t^p_{\text{start}}, t^p_{\text{stop}}] = [t^a_{\text{stop}}, t_{\text{end}}]$ shown in *gray*).

ACKNOWLEDGMENTS

We thank Shenshen Wang and Mason Porter for useful discussions, and we thank members of D.B.'s research group for constructive comments on the manuscript. This work was funded in part by the National Science Foundation Physics of Living Systems under grant 2210316 and in part by the Air Force Office of Scientific Research under grant FA9550-23-1-0713.

AUTHOR CONTRIBUTIONS

D.B. and C.M.-W. designed the research. C.M.-W. developed the theoretical framework, wrote simulation code, performed analytical calculations, and ran simulations. M.A.T. contributed experimental data and wrote the experimental methods section, and C.M.-W. and D.B. wrote the article.

DECLARATION OF INTERESTS

The authors declare no competing interests.

SUPPORTING MATERIAL

Supporting material can be found online at https://doi.org/10.1016/j.bpj.2024.09.006.

REFERENCES

1. Bialek, W. 1987. Physical Limits to Sensation and Perception. *Annu. Rev. Biophys. Biophys. Chem.* 16:455–478. https://doi.org/10.1146/annurev.bb.16.060187.002323.
2. Ó Maoiléidigh, D., and A. J. Ricci. 2019. A Bundle of Mechanisms: Inner-Ear Hair-Cell Mechanotransduction. *Trends Neurosci.* 42:221–236. https://linkinghub.elsevier.com/retrieve/pii/S0166223618303199.
3. Hudspeth, A. J. 2005. How the ear's works work: mechanoelectrical transduction and amplification by hair cells. *C. R. Biol.* 328:155–162. https://linkinghub.elsevier.com/retrieve/pii/S1631069104002975.
4. Geisler, C. D. 1998. From Sound to Synapse: Physiology of the Mammalian Ear. Oxford University Press, New York.
5. Robles, L., and M. A. Ruggero. 2001. Mechanics of the Mammalian Cochlea. *Physiol. Rev.* 81:1305–1352. https://doi.org/10.1152/physrev.2001.81.3.1305.
6. Nuttall, A. L., D. F. Dolan, and G. Avinash. 1991. Laser Doppler velocimetry of basilar membrane vibration. *Hear. Res.* 51:203–213. https://linkinghub.elsevier.com/retrieve/pii/037859559190037A.
7. Yost, W. A., and M. C. Killion. 1997. Chapter 123: Hearing Thresholds. *In* Encyclopedia of Acoustics. 3: Architectural Acoustics, Acoustical Signal Processing, Physiological Acoustics, Psychological Acoustics. Wiley, New York Weinheim.
8. Camalet, S., T. Duke, …, J. Prost. 2000. Auditory sensitivity provided by self-tuned critical oscillations of hair cells. *Proc. Natl. Acad. Sci. USA.* 97:3183–3188. Proceedings of the National Academy of Sciences. https://doi.org/10.1073/pnas.97.7.3183, publisher.
9. Ren, T., W. He, and D. Kemp. 2016. Reticular lamina and basilar membrane vibrations in living mouse cochleae. *Proc. Natl. Acad. Sci. USA.* 113:9910–9915. https://doi.org/10.1073/pnas.1607428113.
10. Gold, T., and J. Gray. 1948. Hearing. II. The physical basis of the action of the cochlea. *Proc. R. Soc. A B.* 135:492–498. https://doi.org/10.1098/rspb.1948.0025.
11. Denk, W., W. W. Webb, and A. J. Hudspeth. 1989. Mechanical properties of sensory hair bundles are reflected in their Brownian motion measured with a laser differential interferometer. *Proc. Natl. Acad. Sci. USA.* 86:5371–5375. https://doi.org/10.1073/pnas.86.14.5371.
12. Martin, P., and A. J. Hudspeth. 1999. Active hair-bundle movements can amplify a hair cell's response to oscillatory mechanical stimuli. *Proc. Natl. Acad. Sci. USA.* 96:14306–14311. Proceedings of the National Academy of Sciences. https://doi.org/10.1073/pnas.96.25.14306, publisher.
13. Martin, P., A. J. Hudspeth, and F. Jülicher. 2001. Comparison of a hair bundle's spontaneous oscillations with its response to mechanical stimulation reveals the underlying active process. *Proc. Natl. Acad. Sci. USA.* 98:14380–14385. publisher: Proceedings of the National Academy of Sciences. https://doi.org/10.1073/pnas.251530598.
14. Nadrowski, B., P. Martin, and F. Jülicher. 2004. Active hair-bundle motility harnesses noise to operate near an optimum of mechanosensitivity. *Proc. Natl. Acad. Sci. USA.* 101:12195–12200.

publisher: Proceedings of the National Academy of Sciences. https://doi.org/10.1073/pnas.0403020101.

15. Ruggero, M. A., and N. C. Rich. 1991. Furosemide alters organ of corti mechanics: evidence for feedback of outer hair cells upon the basilar membrane. *J. Neurosci.* 11:1057–1067. https://doi.org/10.1523/JNEUROSCI.11-04-01057.1991.

16. Dallos, P. 1992. The active cochlea. *J. Neurosci.* 12:4575–4585. https://doi.org/10.1523/JNEUROSCI.12-12-04575.1992.

17. Hudspeth, A. 1997. Mechanical amplification of stimuli by hair cells. *Curr. Opin. Neurobiol.* 7:480–486. https://www.sciencedirect.com/science/article/pii/S0959438897800268.

18. Hudspeth, A. J. 2008. Making an Effort to Listen: Mechanical Amplification in the Ear. *Neuron.* 59:530–545. https://www.ncbi.nlm.nih.gov/pmc/articles/PMC2724262/.

19. Hudspeth, A. J. 2014. Integrating the active process of hair cells with cochlear function. *Nat. Rev. Neurosci.* 15:600–614, , number: 9 Publisher: Nature Publishing Group. https://www.nature.com/articles/nrn3786.

20. Guinan, J. J. 2006. Olivocochlear Efferents: Anatomy, Physiology, Function, and the Measurement of Efferent Effects in Humans. *Ear Hear.* 27:589–607. http://journals.lww.com/00003446-200612000-00001.

21. Maison, S. F., H. Usubuchi, and M. C. Liberman. 2013. Efferent Feedback Minimizes Cochlear Neuropathy from Moderate Noise Exposure. *J. Neurosci.* 33:5542–5552. https://doi.org/10.1523/JNEUROSCI.5027-12.2013.

22. Lin, C.-H. J., and D. Bozovic. 2020. Effects of Efferent Activity on Hair Bundle Mechanics. *J. Neurosci.* 40:2390–2402. https://www.jneurosci.org/content/40/12/2390,publisher:SocietyforNeuroscienceSection:ResearchArticles.

23. Kao, A., S. W. F. Meenderink, and D. Bozovic. 2013. Mechanical Overstimulation of Hair Bundles: Suppression and Recovery of Active Motility. *PLoS One.* 8:e58143. https://journals.plos.org/plosone/article?id=10.1371/journal.pone.0058143,publisher:PublicLibraryofScience.

24. Shlomovitz, R., L. Fredrickson-Hemsing, …, D. Bozovic. 2013. Low Frequency Entrainment of Oscillatory Bursts in Hair Cells. *Biophys. J.* 104:1661–1669. https://www.sciencedirect.com/science/article/pii/S0006349513002877.

25. Martin, P., and A. Hudspeth. 2021. Mechanical Frequency Tuning by Sensory Hair Cells, the Receptors and Amplifiers of the Inner Ear. *Annu. Rev. Condens. Matter Phys.* 12:29–49. https://doi.org/10.1146/annurev-conmatphys-061020-053041.

26. Eguíluz, V. M., M. Ospeck, …, M. O. Magnasco. 2000. Essential Nonlinearities in Hearing. *Phys. Rev. Lett.* 84:5232–5235. https://doi.org/10.1103/PhysRevLett.84.5232, publisher: American Physical Society.

27. Hudspeth, A. J., F. Jülicher, and P. Martin. 2010. A Critique of the Critical Cochlea: Hopf—a Bifurcation—Is Better Than None. *J. Neurophysiol.* 104:1219–1229. publisher: American Physiological Society. https://doi.org/10.1152/jn.00437.2010.

28. Martin, P., and A. J. Hudspeth. 2001. Compressive nonlinearity in the hair bundle's active response to mechanical stimulation. *Proc. Natl. Acad. Sci. USA.* 98:14386–14391. publisher: Proceedings of the National Academy of Sciences. https://doi.org/10.1073/pnas.251530498.

29. Jülicher, F. 2001. Mechanical oscillations at the cellular scale. *Comptes Rendus Acad. Sci. - Ser. IV Phys., Astrophys.* 2:849–860. https://linkinghub.elsevier.com/retrieve/pii/S1296214701012288.

30. Vilfan, A., and T. Duke. 2003. Two Adaptation Processes in Auditory Hair Cells Together Can Provide an Active Amplifier. *Biophys. J.* 85:191–203. https://linkinghub.elsevier.com/retrieve/pii/S0006349503744658.

31. Moreau, L., E. Sontag, and M. Arcak. 2003. Feedback tuning of bifurcations. *Syst. Control Lett.* 50:229–239. https://linkinghub.elsevier.com/retrieve/pii/S0167691103001579.

32. Balakrishnan, J. 2005. Self-tuning to the Hopf bifurcation in fluctuating systems. *J. Phys. Math. Gen.* 38:1627–1652. https://doi.org/10.1088/0305-4470/38/8/001.

33. Bartsch, T. F., F. E. Hengel, …, A. J. Hudspeth. 2019. Elasticity of individual protocadherin 15 molecules implicates tip links as the gating springs for hearing. *Proc. Natl. Acad. Sci. USA.* 116:11048–11056. https://doi.org/10.1073/pnas.1902163116.

34. Dallos, P., X. Wu, …, J. Zuo. 2008. Prestin-Based Outer Hair Cell Motility Is Necessary for Mammalian Cochlear Amplification. *Neuron.* 58:333–339. https://linkinghub.elsevier.com/retrieve/pii/S0896627308001827.

35. Köppl, C., A. Forge, and G. A. Manley. 2004. Low density of membrane particles in auditory hair cells of lizards and birds suggests an absence of somatic motility. *J. Comp. Neurol.* 479:149–155. https://doi.org/10.1002/cne.20311.

36. Moreau, L., and E. Sontag. 2003. Balancing at the border of instability. *Phys. Rev.* 68:020901. https://doi.org/10.1103/PhysRevE.68.020901.

37. Edri, Y., D. Bozovic, and A. Yochelis. 2016. Frequency locking in auditory hair cells: Distinguishing between additive and parametric forcing. *Europhys. Lett.* 116:28002. publisher: EDP Sciences:IOP Publishing and Società Italiana di Fisica. https://doi.org/10.1209/0295-5075/116/28002.

38. Lin, C.-H. J., and D. Bozovic. 2022. Efferent Activity Controls Hair Cell Response to Mechanical Overstimulation. *eNeuro.* 9:publisher: Society for Neuroscience Section: Research Article: New Research. https://www.eneuro.org/content/9/4/ENEURO.0198-22.2022.

39. Howard, J., and A. J. Hudspeth. 1987. Mechanical relaxation of the hair bundle mediates adaptation in mechanoelectrical transduction by the bullfrog's saccular hair cell. *Proc. Natl. Acad. Sci. USA.* 84:3064–3068. https://doi.org/10.1073/pnas.84.9.3064.

40. Martin, P., D. Bozovic, …, A. J. Hudspeth. 2003. Spontaneous Oscillation by Hair Bundles of the Bullfrog's Sacculus. *J. Neurosci.* 23:4533–4548. https://www.jneurosci.org/content/23/11/4533,publisher:SocietyforNeuroscienceSection:Cellular/Molecular.

41. Bozovic, D., and A. J. Hudspeth. 2003. Hair-bundle movements elicited by transepithelial electrical stimulation of hair cells in the sacculus of the bullfrog. *Proc. Natl. Acad. Sci. USA.* 100:958–963. https://doi.org/10.1073/pnas.0337433100.

42. Meenderink, S. W. F., P. M. Quiñones, and D. Bozovic. 2015. Voltage-Mediated Control of Spontaneous Bundle Oscillations in Saccular Hair Cells. *J. Neurosci.* 35:14457–14466. https://doi.org/10.1523/JNEUROSCI.1451-15.2015.

43. Ó Maoiléidigh, D., E. M. Nicola, and A. J. Hudspeth. 2012. The diverse effects of mechanical loading on active hair bundles. *Proc. Natl. Acad. Sci. USA.* 109:1943–1948. publisher: Proceedings of the National Academy of Sciences. https://doi.org/10.1073/pnas.1120298109.

Biophysical Journal, Volume *123*

Supplemental information

Neural control and innate self-tuning of the hair cell's active process

Charles Metzler-Winslow, Martín A. Toderi, and Dolores Bozovic

1 Numerical Simulation Methods

We used the Dormand–Prince method to integrate a discrete-time version of equations 7 and 8 in the main text, with step size $h = 10^{-3}$ time units. For subfigure 3(e) of the main text, we used a variable order, variable step size backward-differentiation formula method to integrate equations 7 and 8.

1.1 Recovery Time

We defined the recovery time t_R as the earliest time after suppression of oscillations at which the sign of either $x(t) - x_{\text{thresh}}$ or $x(t) + x_{\text{thresh}}$ changes for the small threshold displacement parameter $x_{\text{thresh}} = 0.05$. For the recovery from a negative initial value $\mu(0) = \mu_0$, we estimate the recovery time as:

$$t_R(\mu_0) \approx \tau \log(\frac{\mu_0 + \tau(\frac{\alpha}{2} - C)}{\tau(\frac{\alpha}{2} - C)}) \tag{1}$$

The recovery time t_R is shown as a function of the initial value μ_0 in figure 1. For the recovery from a negative value of μ induced through self-tuning by constant additive forcing with duration D, we estimate the recovery time:

$$t_R(D) \approx \tau \log[\frac{e^{-D/\tau}(\alpha - C) - \alpha/2}{\alpha/2 - C}] + t_0 \tag{2}$$

where $t_0 = 7.978$ represents a constant initial time displacement. The recovery time t_R is shown as a function of the constant additive forcing duration D in figure 2.

1.2 Phase-Locked Amplitude

We estimated the phase-locked amplitudes $\mathcal{A} = |\mathcal{F}[x(t)]|(\omega')$ using the discrete Fourier transform, $\mathcal{A}_{\text{numerical}} = |\mathcal{F}_{\text{discrete}}[x(t)]|(\omega_{\text{nearest}})$, where ω_{nearest} denotes the discrete Fourier spectrum frequency closest to the forcing frequency ω'.

1.3 Angular Displacement

We estimated the angular displacement of z as the cumulative sum of the sequence of differences $\mathrm{d}\phi_i = \arg(z(t_{i+1})) - \arg(z(t_i))$ for time step index i.

1.4 Total Power

We estimated the power spectral densities $P[x(t)](\omega')$ using Bartlett's method, $P_{\text{Bartlett}}[x(t)](\omega_{\text{nearest}})$.

1.5 Full Widths at Half Maximum

We estimated the widths of the vector strengths $\mathcal{V} = |\langle e^{i(\phi - \phi')} \rangle_{\text{empirical}}|$ using the `peak_widths` function from the SciPy library. That function accepted the frequency of the peak of the vector strength, ω_{peak}, and executed the following algorithm:

1. Calculate the minimal values $\mathcal{V}^-_{\text{min}}$ and $\mathcal{V}^+_{\text{min}}$ of the vector strength on the intervals $[\omega_-, \omega_{\text{peak}}]$ and $[\omega_+, \omega_{\text{peak}}]$ where $\omega_\pm$ represent the frequencies of the closest peak with a larger height than $\mathcal{V}(\omega_{\text{peak}})$ below and above ω_{peak}, respectively, or the minimum and maximum frequencies if such peaks do not exist.

2. Calculate the peak prominence $P = \mathcal{V}(\omega_{\text{peak}}) - \max(\mathcal{V}^-_{\text{min}}, \mathcal{V}^+_{\text{min}})$.

3. Calculate the evaluation height $\mathcal{V}_{\text{eval}} = \mathcal{V}(\omega_{\text{peak}}) - 0.5 \cdot P$.

4. Calculate the frequencies $\omega^\pm_{\text{eval}}$ at which $\mathcal{V}(\omega^\pm_{\text{eval}}) = \mathcal{V}_{\text{eval}}$ using linear interpolation between the closest points of $\mathcal{V}(\omega')$ above and below $\mathcal{V}_{\text{eval}}$ on each side of the peak.

5. Return the width $\Delta\omega'_{\mathcal{V}_{\max/2}} = \omega^+_{\text{eval}} - \omega^-_{\text{eval}}$.

2 Choice of Parameter Values

The values of the fixed parameters ω and β that appear in equation 8 in the main text, as well as the parameters τ, α, and C in equation 7, affect the bundle state and control parameter dynamics significantly. The ranges of acceptable values for these parameters are constrained by conditions imposed by the modeling objectives.

The ranges of values that the parameters α and C can take are constrained by the condition that the steady state value of $\mu(t)$ must be positive in the case of zero additive forcing (so that spontaneous oscillations occur) and negative in the case of strong additive forcing (so that spontaneous oscillations are suppressed.) In the zero forcing case, the approximate steady state value of μ is $\tau(C - \frac{\alpha}{2})$, and the approximate value during overstimulating forcing is $\tau(C-\alpha)$, so the constraint is $\alpha/2 < C < \alpha$.

The intrinsic frequency ω and nonlinear parameter $\beta = b' + ib''$ are connected to the parameters τ, C, and α through the condition that the spontaneous oscillation frequency is positive, $\mathrm{d}_t \arctan(y/x) > 0$. The approximate steady-state value of the oscillation frequency is $\omega - (b''/b')\mu_{\text{steady-state}}$, so the constraint on the intrinsic frequency is $\omega > \tau b''(C - \frac{\alpha}{2})/b'$.

3 Parametric Forcing of Spontaneous Oscillations

The parametric forcing function $F_p(t)$ and control parameter term $\mu \mathbf{1}_{F_p \leq 0}(t)$ are shown in figure 3 for the case of zero additive forcing, $F_a = 0$. Since the control parameter self-tuning equation imposes negative regulation on μ as a function of position $x(t)$, the steady-state value of the control parameter is larger immediately after the cessation of parametric forcing. An example of the negative effect of parametric forcing on phase-locking to sinusoidal forcing is shown in figure 4; the state function $x(t)$ is shown for three values of parametric forcing amplitude, chosen to be fractions of the steady-state average value of the oscillations of the control parameter generated by the self-tuning equation, $\bar{\mu} = \langle \mu_{\text{self-tuning active}}(t) \rangle$. An example of the decrease in total response to sinusoidal forcing and blunting of frequency sensitivity (the widening of the response curves $\mathcal{A}(\omega')$) as a result of parametric forcing is shown in figure 5.

4 Self-Tuning and Sinusoidal Additive Forcing

Spontaneous oscillations produced by a Hopf oscillator endowed with the self-tuning equation for the control parameter $\mu(t)$ are similar in steady state to oscillations produced by a Hopf oscillator with a constant value of μ chosen to be the steady-state average value of the self-tuning control parameter oscillations, $\mu_{\text{self-tuning inactive}} = \bar{\mu}$. The responses of a self-tuned Hopf oscillator and a Hopf oscillator with $\mu = \mu_{\text{self-tuning inactive}}$ to sinusoidal forcing are shown in figure 6. The phase-locked amplitude $\mathcal{A}(F)$ is shown for both oscillators as a function of the amplitude of sinusoidal forcing at the common frequency of spontaneous oscillation in figure 7; it is clear that the oscillators display approximately identical response power laws.

Without the control parameter self-tuning equation, a Hopf oscillator remains on the oscillatory side of the bifurcation during large-amplitude overstimulating forcing, so the recovery time $t_R = 0$.

5 Self-Tuning and Overstimulating Forcing

Consistent with experimental observations of the properties of spontaneous hair bundle oscillations immediately after the recovery from overstimulating forcing, we found that, following large-amplitude constant forcing, post-recovery oscillations of a Hopf oscillator endowed with control parameter self-tuning grew in amplitude, shrunk in frequency, and grew in the per-cycle ratio of the length of the interval above the zero point of x to the length of the cycle (this quantity has been used as an estimate of the open probability of mechanotransduction channels.) These changes in the properties of post-recovery oscillations are shown in figures 8, 9, and 10.

6 Parametric Forcing of τ

We applied parametric forcing to the parameter μ by modifying the self-tuning equation to the following:

$$\frac{\mathrm{d}\mu}{\mathrm{d}t} = -\frac{\mu}{\tau F_p} - \frac{\alpha}{1 + e^{-\gamma x}} + C \tag{3}$$

Where the parametric forcing function $F_p(t) = \Gamma(\Theta(t - t^p_{\text{on}}) - \Theta(t - t^p_{\text{off}}))$ for forcing amplitude Γ. According to equation 1, the recovery time $t_R(\tau)$ is a monotonically increasing function of time constant τ, so parametric forcing with amplitude $\Gamma \ll 1$ of the time constant during the interval following cessation of overstimulating constant additive forcing has the effect of reducing the recovery time so that recovery is almost instantaneous. Such parametric forcing also causes the amplitude of spontaneous oscillations to decrease (the steady-state time average amplitude $\langle A \rangle \approx \sqrt{-\tau(C - (\alpha/2))/b'}$) and frequency to increase ($\langle \dot{\phi} \rangle \approx \omega - \tau b''(C - (\alpha/2))/b'$).

7 Denoising of Experimental Data

Experimental data were denoised using a first-order digital Butterworth filter with critical frequency 50 Hz calculated as cascaded biquadratic sections.

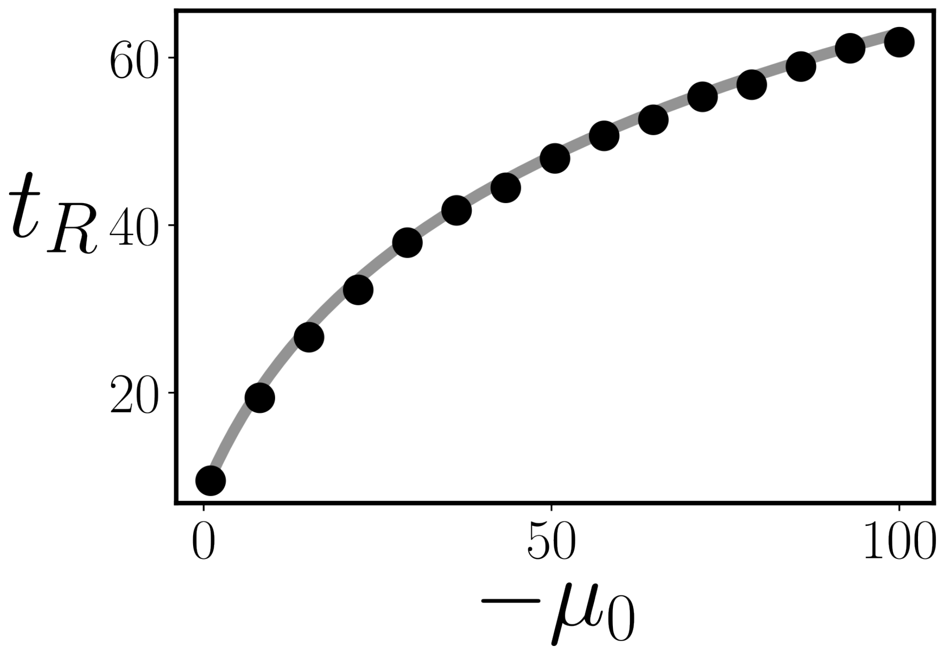

Figure 1: Duration $t_R(\mu_0)$ (black disks) preceding recovery of spontaneous oscillations from initial negative (quiescent) value of the control state parameter μ. The estimate defined by equation 1 is shown in gray.

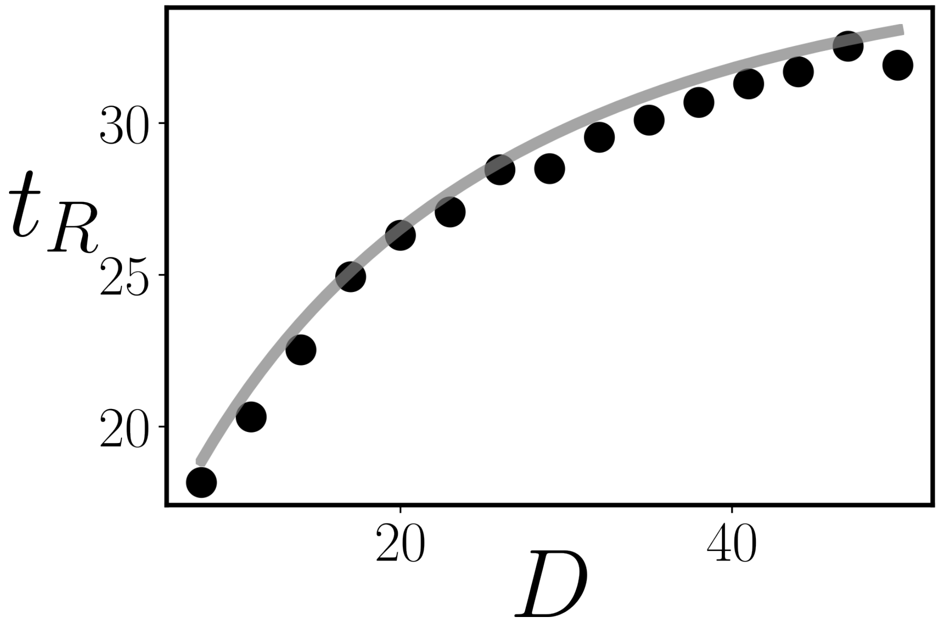

Figure 2: Duration $t_R(D)$ (black disks) preceding recovery of spontaneous oscillations from overstimulating constant forcing $F = [\Theta(t - t^a_{\text{start}}) - \Theta(t - t^a_{\text{stop}})]F$ with strength $F = 60$ and duration $D = t^a_{\text{stop}} - t^a_{\text{start}}$. The estimate defined by equation 2 is shown in gray.

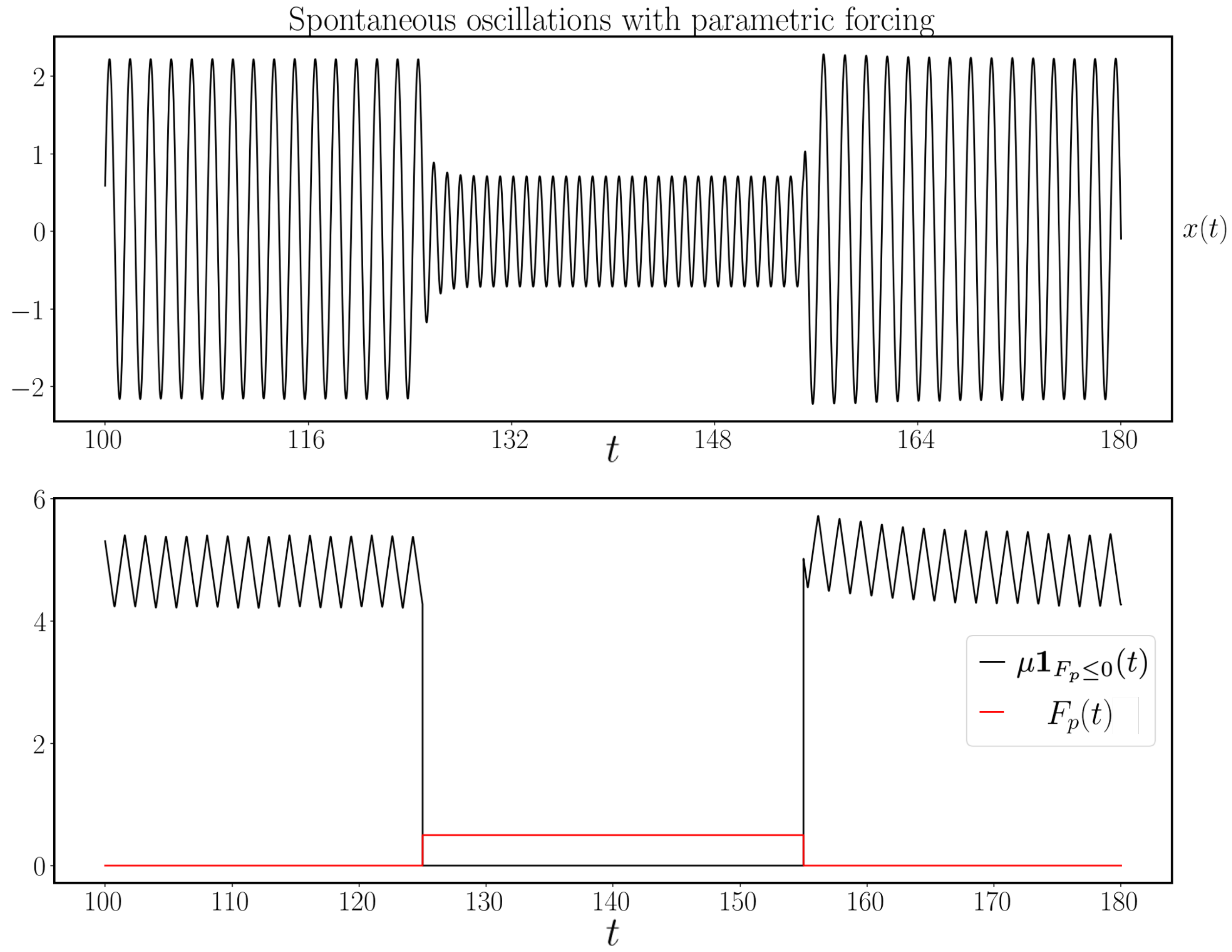

Figure 3: State function $x(t)$ (shown in black) for a Hopf oscillator with parametric forcing. Control parameter term $\mu(t)\mathbf{1}_{F_p\leq 0}(t)$ shown in lower panel (black curve) with rectangular parametric forcing function $F_p(t)$ (red curve.)

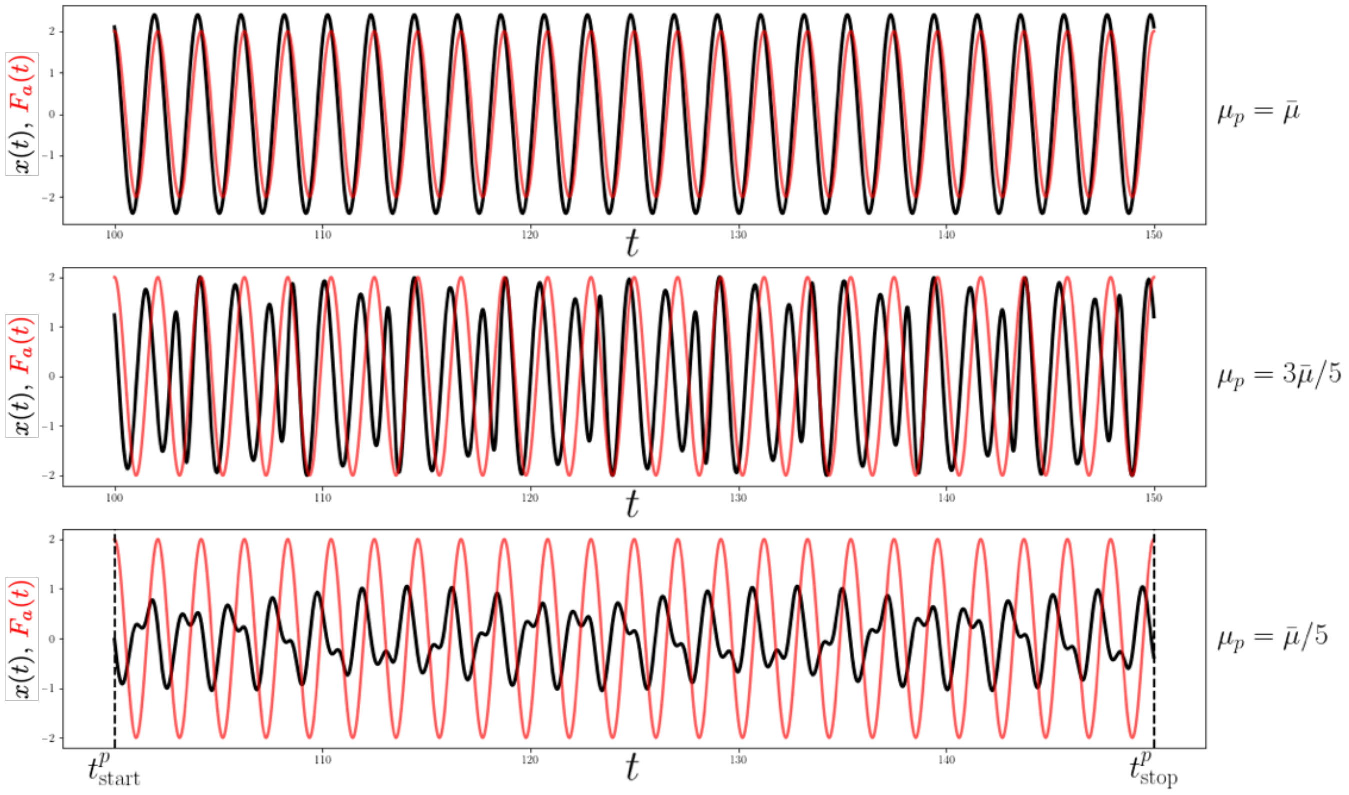

Figure 4: Effect of parametric forcing on phase-locking. Figure: response of state function $x(t)$ (shown in black) to identical additive forcing $F_a(t)$ (shown in red) for three values of parametric forcing amplitude μ_p.

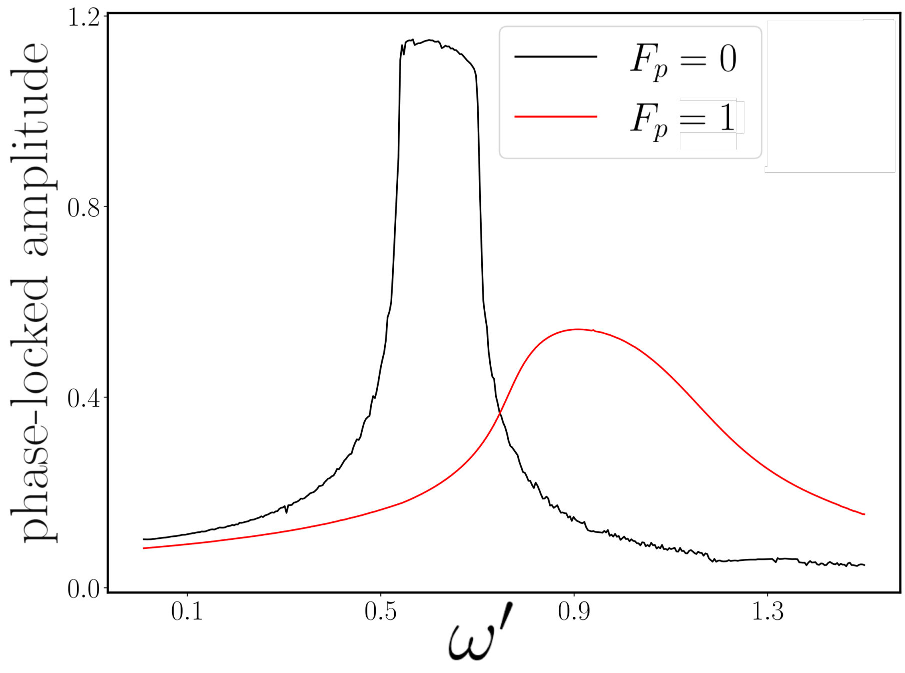

Figure 5: Parametric forcing causes the total phase-locked forcing response of a Hopf oscillator to sinusoidal forcing to decrease and widens the range of frequencies to which a Hopf oscillator is sensitive. Figure: normalized Fourier amplitudes $\mathcal{A}(\omega') = |\mathcal{F}[x(t)]|(\omega')$ representing 1:1 phase-locking to sinusoidal forcing $F_a = e^{i\omega' t}$ shown in the case of no parametric forcing and constant parametric forcing $F_p = \mu_p$ with amplitude $\mu_p = 1$.

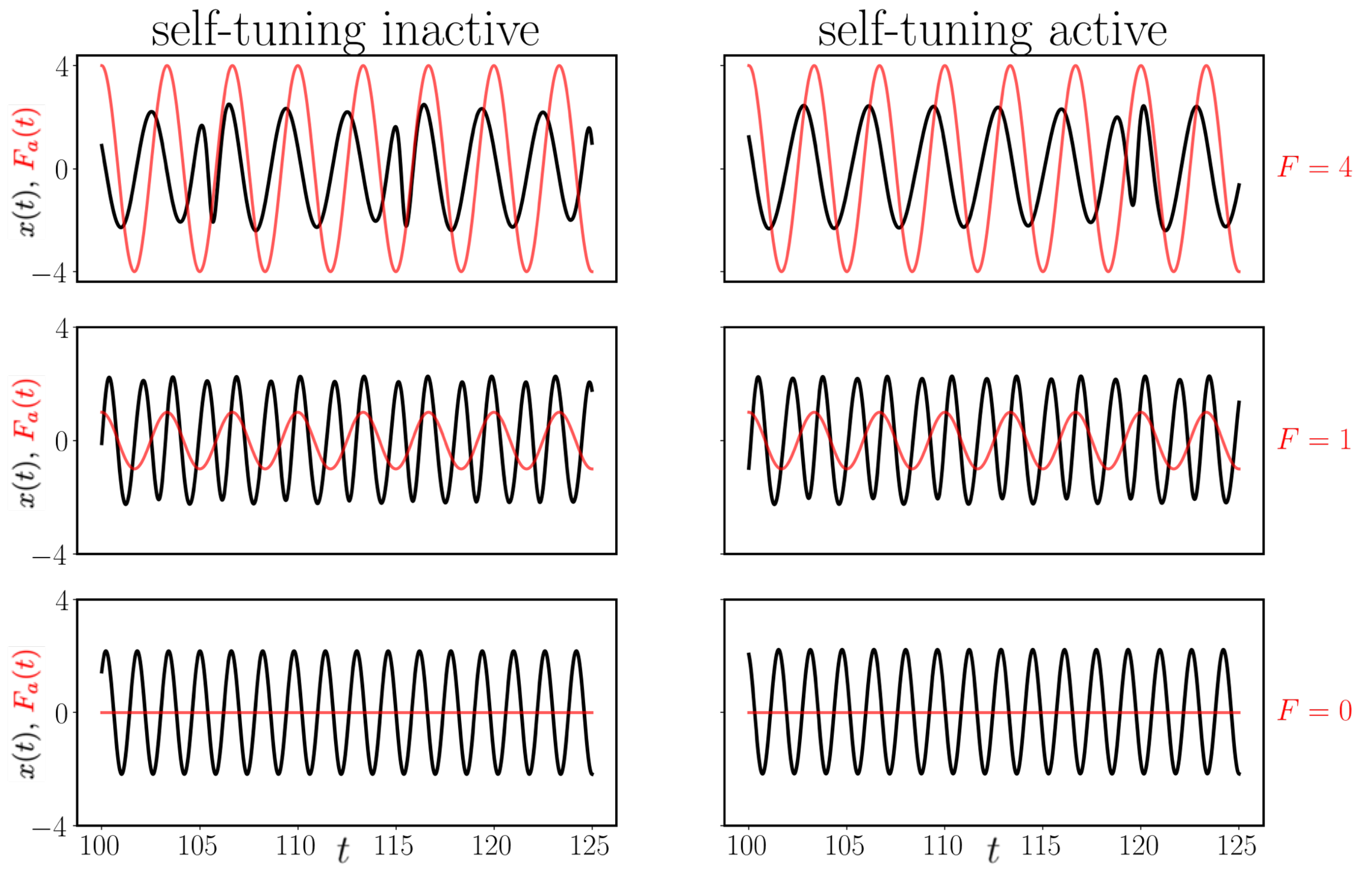

Figure 6: State functions $x(t) = \mathrm{Re}[z(t)]$ for (1) a Hopf oscillator with control parameter self-tuning feedback (left column) and (2) a Hopf oscillator with $\mathrm{d}\mu/\mathrm{d}t = 0$ and $\mu = \langle\bar{\mu}_{\mathrm{ss}}\rangle_{\mathrm{sample}}$, the average over additive forcing amplitudes of the steady-state average of the control parameter $\mu(t)$ subject to self-tuning (as in the right-hand column.) The amplitude of additive forcing $F_a = Fe^{i\omega' t}$ with $\omega' = 0.3$ (red solid lines) increases up from the bottom row. For both oscillators, the nonlinear parameter $\beta = -1 - 0.5i$ and the frequency parameter $\omega = 2\pi$.

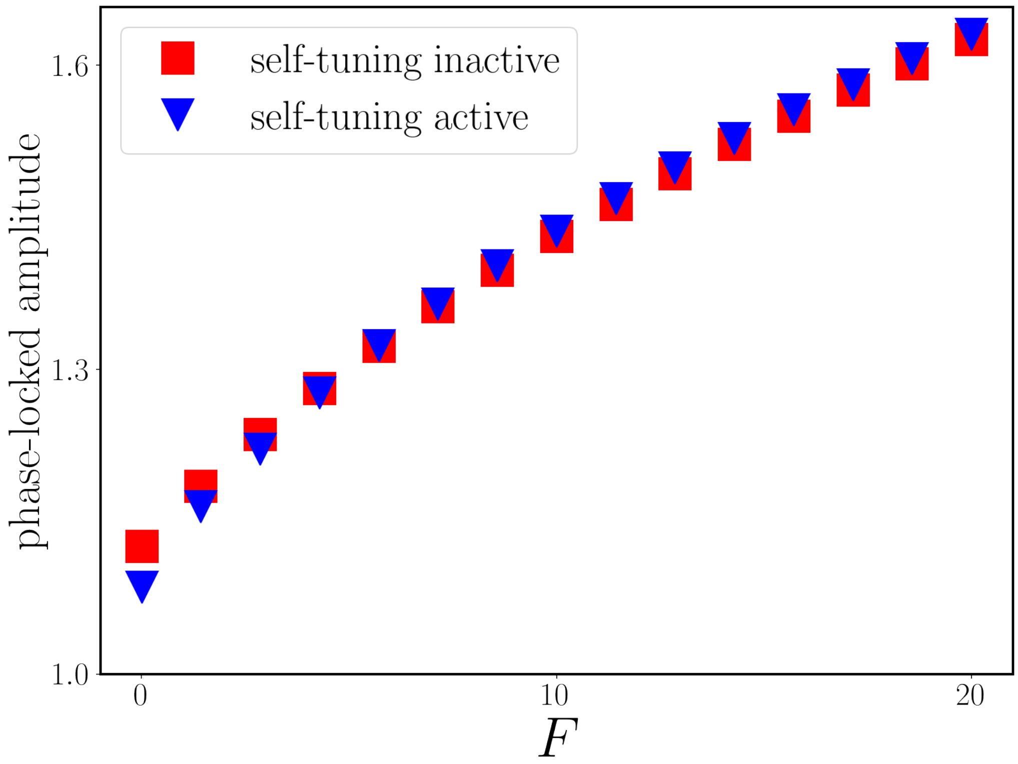

Figure 7: Phase-locked amplitude as a function of amplitude of additive sinusoidal forcing $F_a(t) = Fe^{i\omega' t}$ with $\omega' = \dot{\phi}$, the intrinsic frequency, for Hopf oscillators with and without control parameter self-tuning feedback.

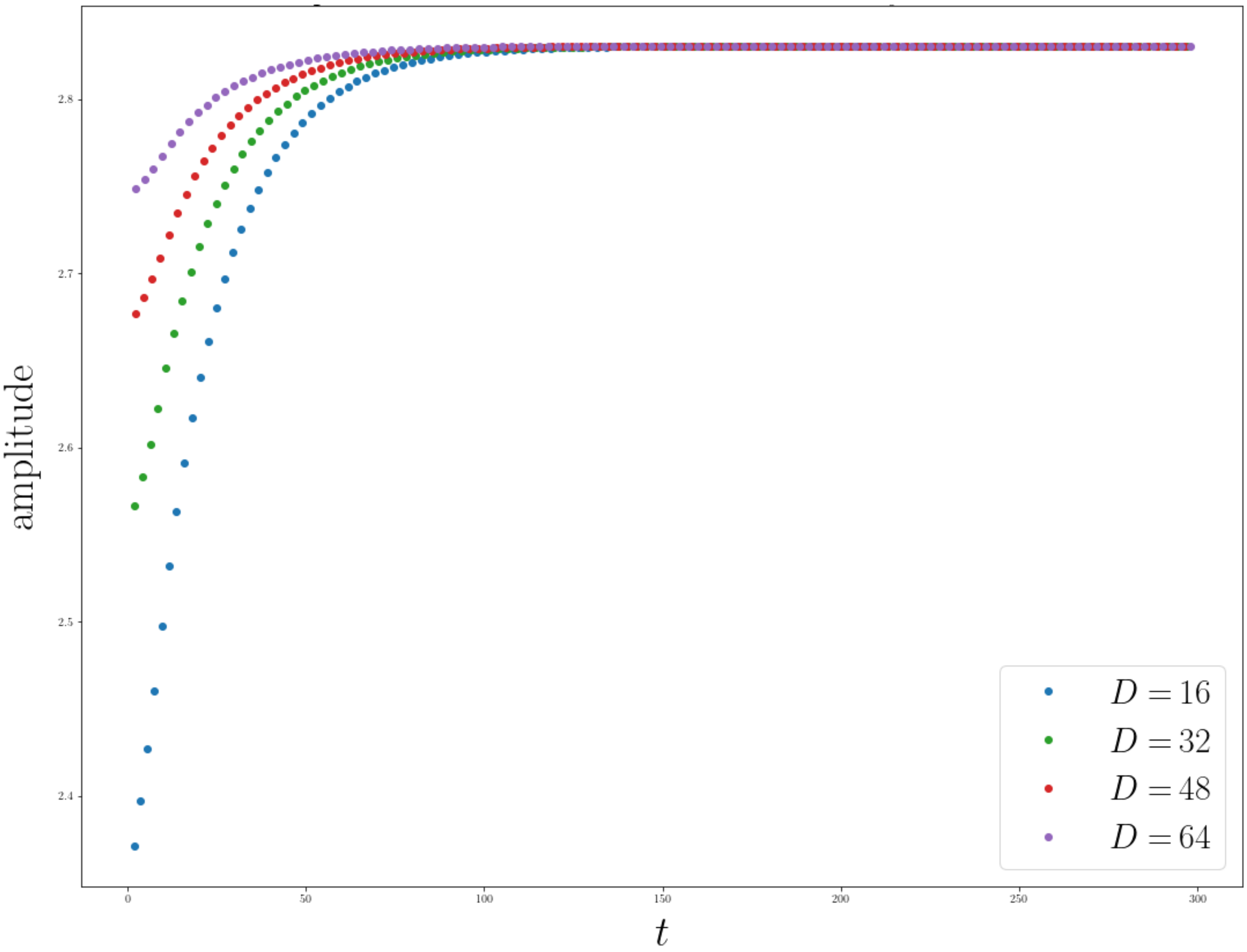

Figure 8: The effect of self-tuning on spontaneous oscillation amplitude following the cessation of overstimulating forcing for four forcing durations $D = t^a_{\text{start}} - t^a_{\text{stop}}$. Amplitude is measured as the moving mean of $(\langle x_{\text{above}} \rangle - \langle x_{\text{below}} \rangle)/2$ where $\langle x_{\text{above, below}} \rangle$ represent the empirical means within each cycle i of the values of x greater than and less than the value x_{midline} corresponding to the minimum of the Gaussian kernel density estimate of $x(t)$, respectively.

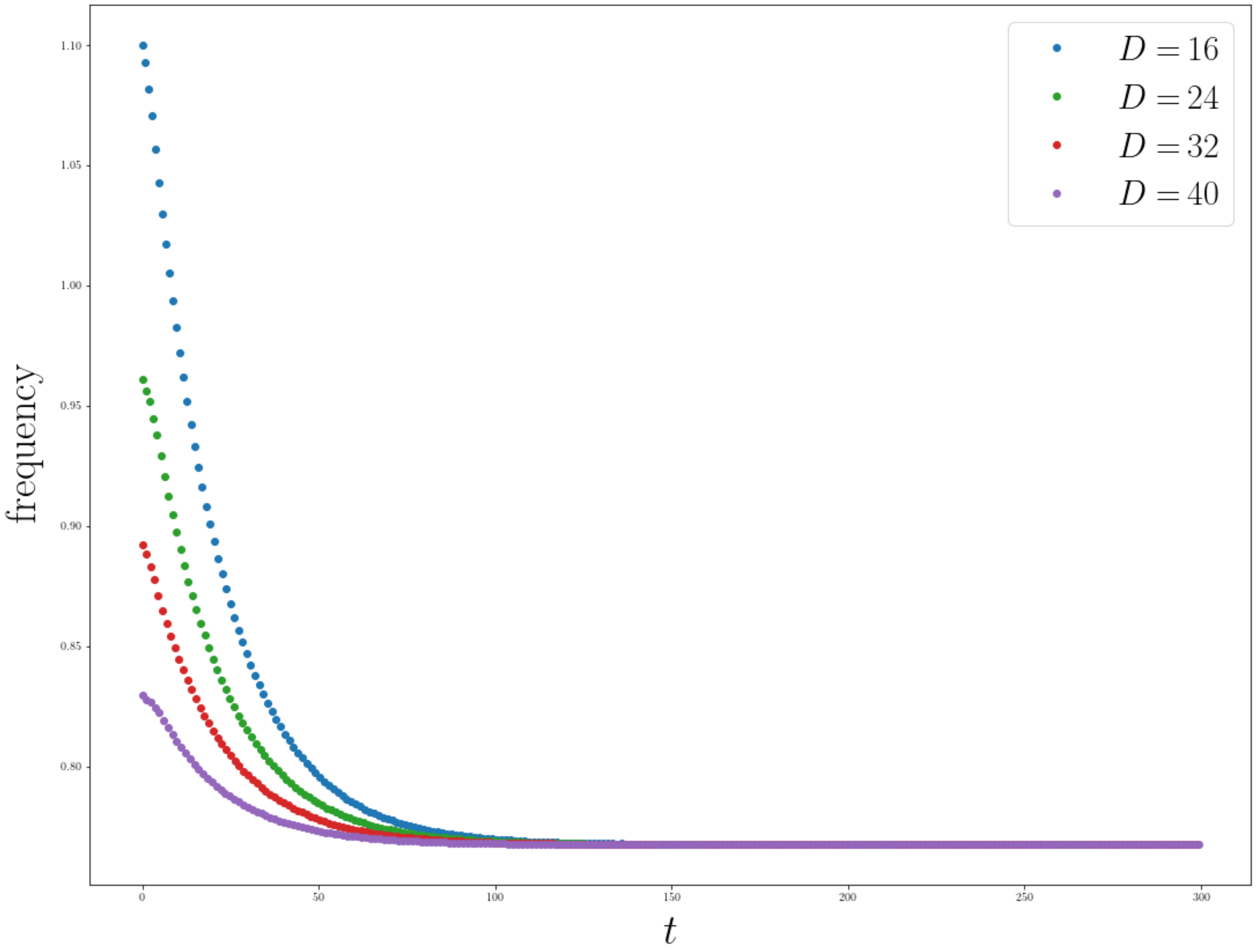

Figure 9: The effect of self-tuning on spontaneous oscillation frequency, measured as the moving mean of $f(t_i) = 1/T_i$ for period T_i of cycle i, following the cessation of overstimulating forcing.

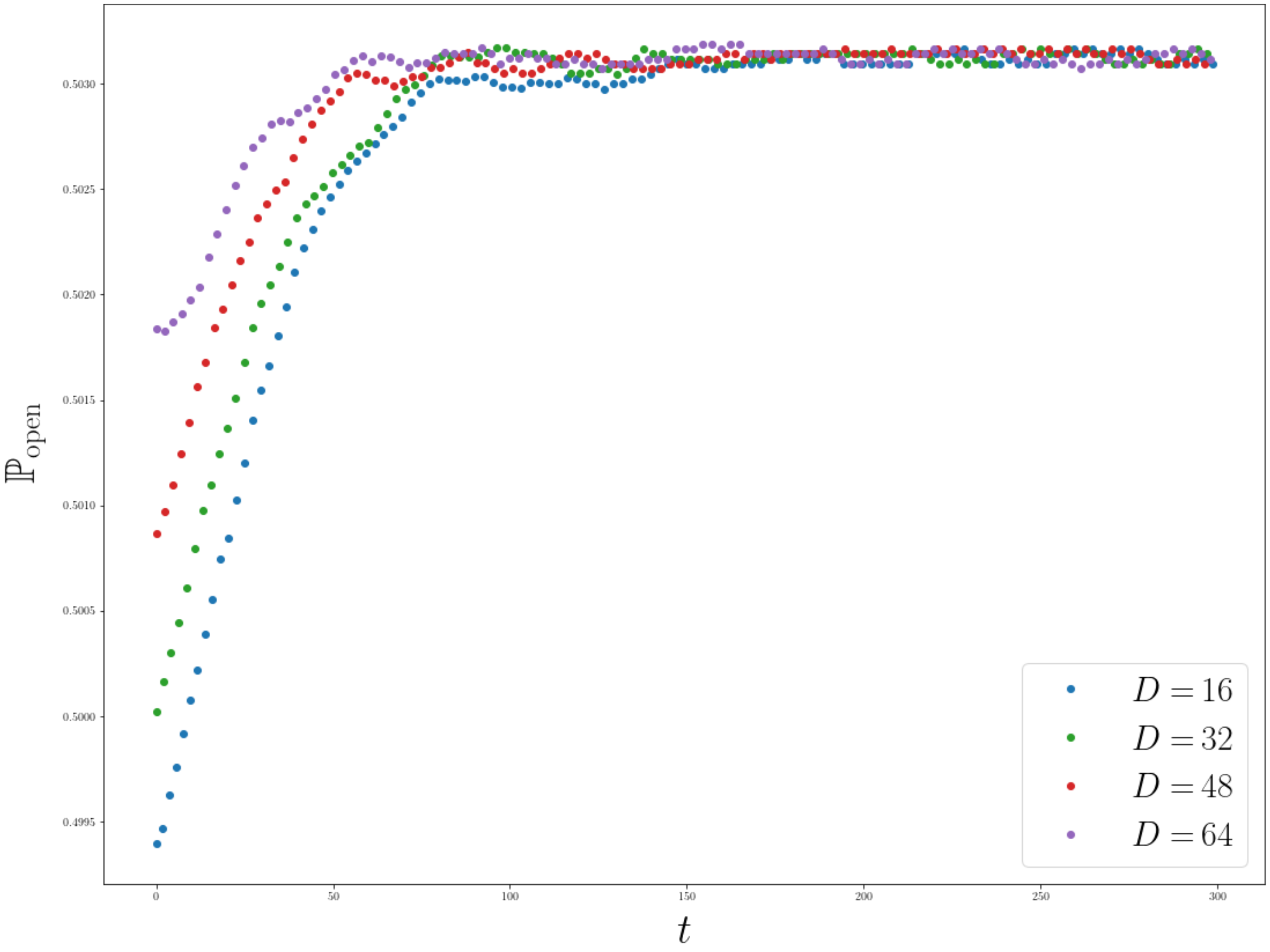

Figure 10: The effect of self-tuning on open probability, measured as the moving mean of the ratio within each cycle i of the number of samples for which all values of x are greater than x_{midline} to the total number of cycles in the cycle, following the cessation of overstimulating forcing.
